# XoxF and the Calvin-Benson cycle mediate lanthanide-dependent growth on methanol in *Bradyrhizobium* and *Sinorhizobium*

**DOI:** 10.1128/aem.01304-25

**Published:** 2025-10-21

**Authors:** Charlotte R. Mineo, Jerry Jiang, N. Cecilia Martinez-Gomez

**Affiliations:** 1 Department of Plant & Microbial Biology, University of California, Berkeley 1438, Berkeley, California, USA; The University of Tennessee Knoxville, Knoxville, Tennessee, USA

**Keywords:** methylotrophy, rare earth elements, CBB cycle

## Abstract

Nodule-forming bacteria play crucial roles in plant health and nutrition by providing fixed nitrogen to leguminous plants. Despite the importance of this relationship, how nodule-forming bacteria are affected by plant exudates and soil minerals is not fully characterized. Here, the effects of plant-derived methanol and lanthanide metals on the growth of nitrogen-fixing *Rhizobiales* are examined. Prior work has demonstrated that select *Bradyrhizobium* strains can assimilate methanol only in the presence of lanthanide metals; however, the pathway enabling assimilation remains unknown. In this study, we characterize *Bradyrhizobium diazoefficiens* USDA 110, *Bradyrhizobium* sp. USDA 3456, and *Sinorhizobium meliloti* 2011 to determine the pathways involved in methanol metabolism in previously characterized strains, other clades of *Bradyrhizobium*, and the more distantly related *Sinorhizobium*. Based on genomic analyses, we hypothesized that methanol assimilation in these organisms occurs via the lanthanide-dependent methanol dehydrogenase XoxF, followed by oxidation of formaldehyde via the glutathione-linked oxidation pathway, subsequent oxidation of formate via formate dehydrogenases, and finally assimilation of CO_2_ via the Calvin-Benson-Bassham (CBB) cycle. Transcriptomics revealed upregulation of the aforementioned pathways in *Bradyrhizobium* sp. USDA 3456 during growth with methanol. Enzymatic assays demonstrated increased activity of the glutathione-linked oxidation pathway and formate dehydrogenases in all strains during growth with methanol compared to succinate. ^13^C-labeling studies confirmed the presence of CBB intermediates and label incorporation during growth with methanol. Our findings provide multiple lines of evidence supporting the proposed XoxF-CBB pathway and, combined with genomic analyses, suggest that this metabolism is widespread among *Bradyrhizobium* and *Sinorhizobium* species.

**IMPORTANCE:**

Nitrogen-fixing soil bacteria such as *Bradyrhizobium* and *Sinorhizobium* promote plant growth while reducing dependence on artificial, energy-intensive fertilizers. Numerous studies have attempted to increase bacterial nitrogen fixation and colonization of plant tissues by identifying the micronutrients and plant exudates that promote successful symbiotic relationships. Among the compounds encountered by rhizobacteria, lanthanides have received little attention, despite reports that plant growth is affected by the presence of lanthanides. We characterized three agriculturally relevant *Bradyrhizobium* and *Sinorhizobium* strains, demonstrated that they gain the capacity to utilize methanol when lanthanides are present, and experimentally determined the pathway by which this metabolism occurs. This study provides a foundation for understanding the lanthanide-dependent metabolism of *Bradyrhizobium* and *Sinorhizobium,* which may influence their physiology and abundance in the environment.

## INTRODUCTION

Nitrogen-fixing soil bacteria are crucial for global nitrogen cycles, high-yield crop production, and reduced fertilizer needs (1). Microbes supply approximately 50% of the fixed nitrogen consumed by crops, with the rest coming from energy-intensive artificial fertilizers (2). Diverse bacteria have been shown to fix nitrogen both in free-living forms and as differentiated cells, which fix N_2_ within the low-oxygen environment present in specialized plant root organs called nodules (3). *Bradyrhizobium* and *Sinorhizobium* are two genera of nodule-forming bacteria that colonize a range of legume species in a strain-dependent manner, but are especially well-known for colonizing soybean and alfalfa, respectively (4).

*Bradyrhizobium*, *Sinorhizobium*, and other nodule-forming bacteria encounter diverse substrates as they migrate between the bulk soil and the carbon-rich rhizosphere, colonize root tissues, and fix N_2_ as bacteroids in root nodules (5). One such substrate is methanol, an abundant volatile carbon source in plant environments due to the activity of pectin methylesterases, which release methanol from both live plant tissues and plant detritus (6, 7). In above-ground plant tissues, methanol has been shown to drive colonization by allowing bacteria capable of using methanol as a sole source of carbon and energy, methylotrophs, to out-compete non-methylotrophic strains (8, 9). Similar mechanistic studies have not yet been conducted in soils or with N_2_-fixing bacteria, although it is known that *Bradyrhizobium* assimilate methanol *in situ* in grassland soils and the rhizosphere (10).

Lanthanide (Ln) metals are required cofactors for methanol metabolism in various plant-associated bacteria, including *Methylobacterium* and *Bradyrhizobium* (11–13). Lns are involved in methanol metabolism as cofactors in certain pyrroloquinoline quinone (PQQ) methanol dehydrogenases (PQQ-MDH) that oxidize methanol to formaldehyde. The Ln-dependent PQQ-MDH XoxF was first shown to be functional and require Lns in 2011 (14). To facilitate the binding of Lns, XoxF contains an additional Asp in the active site compared to the homologous, Ca-dependent PQQ-MDH MxaF. This residue is essential for Ln binding and catalysis and is used to predict Ln dependency (15). Lns are introduced to soil through fertilizers (16–18) and can therefore influence the physiology of soil-dwelling microbes such as *Bradyrhizobium* and *Sinorhizobium*.

The activity of PQQ-MDHs, such as XoxF and MxaF, produces formaldehyde, a reactive electrophile that forms cross-links with DNA and proteins (19). Multiple pathways exist to oxidize formaldehyde and generate NAD(P)H, but the tetrahydromethanopterin (H_4_MPT) and glutathione (GSH)-linked oxidation pathways are most common (20). *Methylobacterium, Hyphomicrobium*, and many methanotrophs rely on the H_4_MPT pathway for formaldehyde oxidation (21, 22), while the GSH-linked pathway has been shown to play a supportive role in formaldehyde oxidation in *Methylobacterium aquaticum* 22A and *Rhodobacter sphaeroides* (23, 24), and is essential for growth with methanol in *Paracoccus denitrificans* (25). These pathways use different carbon carriers (H_4_MPT or glutathione) but similar reactions: both pathways covalently link formaldehyde to their respective carbon carrier, then oxidize the formyl group, and finally release the formyl carbon from the carrier to recycle the carrier and generate formate. Formate can be further oxidized by formate dehydrogenases (FDHs) to generate CO_2_ and additional NAD(P)H that fuel cellular processes and assimilatory cycles (26).

Carbon from methanol can be assimilated at several steps—formaldehyde, formate, or CO_2_—using diverse pathways, but the specific pathways employed by *Bradyrhizobium* and *Sinorhizobium* remain unknown. In type I methylotrophy, Gamma- and Betaproteobacteria use the ribulose monophosphate (RuMP) pathway to incorporate formaldehyde (27). Type II methylotrophs assimilate carbon at the level of formate. This group includes mostly Alphaproteobacteria, such as the model methylotroph *Methylobacterium extorquens* AM1 (*Me.* AM1). In *M. extorquens* AM1, approximately 50% of formate is oxidized to CO_2_ to generate NAD(P)H, the remaining 50% is incorporated into the serine cycle (21, 27, 28), and the ethylmalonyl-CoA (EMC) pathway is used to regenerate glyoxylate (29, 30).

As an alternative to the RuMP pathway and serine cycle, all carbon from methanol can be fully oxidized to CO_2_ and assimilated via the Calvin-Benson-Bassham (CBB) cycle. Operating the CBB cycle may appear inefficient due to the high energy requirements for CO_2_ fixation. However, use of the CBB cycle for growth with methanol has been validated in nitrogen-fixing Alphaproteobacteria such as *Beijerinckia mobilis* and *Xanthobacter flavus*, as well as the verrucomicrobial methanotroph *Methylacidiphilum fumariolicum* SolV, and the Gammaproteobacteria methanotroph *Methylococcus capsulatus* Bath (31–35). Keltjens et al. and Huang et al. have suggested that *Bradyrhizobium* and *Sinorhizobium* may be able to assimilate methanol using the CBB cycle, but this metabolism has yet to be experimentally validated (36, 37).

Despite their agricultural importance and ability to assimilate methanol *in situ* in grassland soils (10), very few studies have characterized methanol metabolism or Ln dependency among the *Bradyrhizobium* and *Sinorhizobium*. In separate *in vitro* studies, *Bradyrhizobium diazoefficiens* USDA 110 (*Bd*. 110) and *Bradyrhizobium* sp. Ce-3 were shown to grow with methanol only when light Lns (lanthanum, cerium, praseodymium, or neodymium) were present, *mxaF* homologs were not identified in these strains, and growth was linked to the activity of XoxF (12, 13). To our knowledge, growth with methanol has not been reported among the *Sinorhizobium,* despite the *in vitro* characterization of a XoxF homolog from *Sinorhizobium meliloti* 5A14, which requires light Lns for activity (36). Notably, *Sinorhizobium meliloti* genes for methanol oxidation are located directly upstream of genes of the CBB cycle, such as *cbbL* which encodes the large subunit of RuBisCO, on the symbiotic megaplasmid pSymB (38). Growth with formate has been observed in *Sinorhizobium* and is known to be dependent on the presence of the CBB cycle and an NAD^+^-dependent molybdenum-containing formate dehydrogenase, Fds (39).

**Fig 1:**
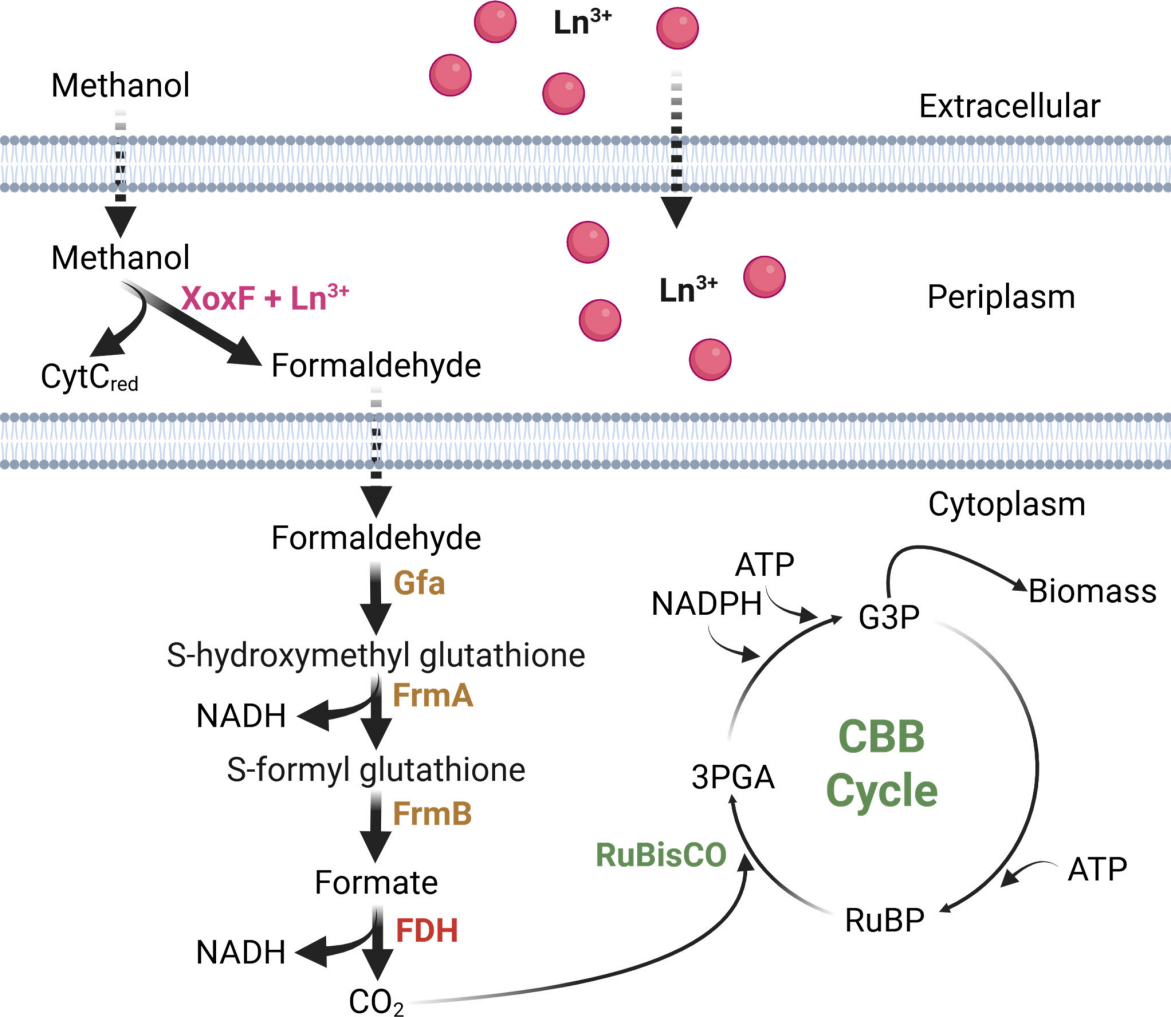
Schematic overview of the proposed XoxF-CBB pathway for methanol metabolism in *Bradyrhizobium* and *Sinorhizobium*. La^3+^ is transported into the cell and complexes with apo-XoxF. XoxF (pink) oxidizes methanol to formaldehyde in the periplasm. Formaldehyde is oxidized to formate in the cytoplasm by the multi-step GSH-linked oxidation pathway (brown). Formate is oxidized to CO_2_ by an NAD^+^-dependent formate dehydrogenase (red). CO_2_ can be assimilated via the Calvin-Benson cycle (green). For clarity, only the most relevant steps of the CBB cycle are depicted. Dashed lines represent hypothesized steps based on similarity with other systems. Solid lines show experimentally supported steps from this study. XoxF, lanthanide-dependent methanol dehydrogenase; Gfa, glutathione-dependent formaldehyde activating enzyme; FrmA, S-(hydroxymethyl)glutathione dehydrogenase; FrmB, S-formylglutathione hydrolase; FDH, NAD^+^-dependent formate dehydrogenase; 3PGA, 3-phosphoglycerate; G3P, glyceraldehyde-3-phosphate; RuBP, ribulose 1,5-bisphosphate. Created in https://BioRender.com.

Based on bioinformatic analyses, we hypothesize that *Bradyrhizobium* and *Sinorhizobium* oxidize methanol using XoxF, metabolize formaldehyde via the GSH-linked oxidation pathway, produce CO_2_ using FDHs, and assimilate CO_2_ using the CBB cycle (Fig. 1) (hereafter referred to as the XoxF-CBB pathway). Transcriptomic, proteomic, metabolomic, and biochemical studies show that the XoxF-CBB pathway in *Bradyrhizobium* sp. USDA 3456 (*Bs*. 3456) is operational. Additionally, we use proteomics and biochemical assays to confirm that the XoxF-CBB pathway operates in another species, *Bd*. 110, and the more distantly related *Sinorhizobium meliloti* 2011 (*Sm.* 2011). This is the first report of Ln use and methanol assimilation by *Sinorhizobium*. Bioinformatic analyses suggest that methanol metabolism via the XoxF-CBB pathway is widespread among nitrogen-fixing *Rhizobiales*, including *Bradyrhizobium, Sinorhizobium,* and *Mesorhizobium*. This work reveals overlooked methanol metabolism pathways and new Ln-dependent species, allowing for a better understanding of the effects of methanol and Ln on nitrogen-fixing bacteria and agricultural settings.

## MATERIALS AND METHODS

### Media and growth conditions

All strains were grown at 30°C with orbital shaking at 200 rpm in an Innova S44i incubator shaker (Eppendorf). Strains and plasmids used in this study are listed in Table 1. Pre-cultures of *Bs*. 3456, *Bd*. 110, *Sm.* 2011, and *Me* AM1 were grown in *Methylobacterium* PIPES (MP) defined minimal media (40) supplemented with either 1 g/L of gluconate, arabinose, and yeast extract or 15 mM succinate. When preparing growth curves or larger cultures for assays, pre-cultures were spun down at 2,000 × *g* for 10 minutes at 22°C, washed in MP without carbon sources, and used to inoculate at a starting OD_600_ of 0.05–0.1. Growth was monitored at OD_600_ with either 50 mM methanol or 15 mM succinate with 10 µM LaCl_3_ when indicated. Experiments for Fig. S1 were conducted with the indicated Ln species in 650 µL cultures in transparent 48-well plates (Corning), incubated at 30°C with orbital shaking at 548 rpm, with OD_600_ readings every 60 minutes using a Synergy HTX plate reader (Agilent). Otherwise, aerobic cultivation for growth curves occurred with 5 mL of culture in sterile round-bottom 14 mL polypropylene tubes (Falcon). Experiments in varying oxygen conditions were performed in Balch-style tubes covered either with sterile tinfoil (unsealed) or plugged with blue stoppers (sealed condition). Growth was monitored by measuring OD_600_ using either the Ultrospec 10 (Amersham Biosciences) for polypropylene tubes or the Thermo Scientific GENESYS version 20 spectrophotometer for Balch-style tubes.

**TABLE 1:** Strains, plasmids, and primers used in this study

Resource	Description	Reference
Strains		
*Escherichia coli* 10β	Cloning – electrocompetent strain	Invitrogen
*Escherichia coli* S17-1	Cloning – conjugation strain	New England BioLabs
*Methylobacterium extorquens* AM1	Wild-type *Me.* AM1	(41)
*Bradyrhizobium diazoefficiens* USDA 110	Wild-type *Bd.* 110	(42)
*Bradyrhizobium* sp. USDA 3456	Wild-type *Bs.* 3456	(43)
*Sinorhizobium meliloti* 2011	Wild-type *Sm*. 2011	(44)
*Methylobacterium extorquens* AM1 Δ*mxaF*	*Me.* AM1 Δ*mxaF*	(41)
*Bradyrhizobium diazoefficiens* USDA 110 Δ*cbbS*	*Bd.* 110 Δ*cbbS*	This study
Plasmids		
pCM433KanT	SacB allelic exchange backbone; Km^R^	(45)
pCRM031	SacB allelic exchange donor *cbbS*; Km^R^	This study
Primers used to generate plasmid pCRM031		
Backbone	YR21: ATGTGCAGGTTGTCGGTGTC YR22: TGGTAACTGTCAGACCAAGTTTACTC	This study
5´ Flank	CRM148: ACTTGGTCTGACAGTTACCACGCCAAGGAGCTCGGTTCG CRM149: GCCAAGGAAACGGAGCAGGGACACGAAGTCCGGCGTGTC	This study
3´ Flank	CRM150: CACGCCGGACTTCGTGTCCCTGCTCCGTTTCCTTGG CRM151: GACACCGACAACCTGCACATGCGAACGGAAGCCGGGATTG	This study

### Biochemical assays

Fifty milliliter cultures were grown in sterile 250 mL flasks with 10 µM LaCl_3_ and either 50 mM methanol or 15 mM succinate. All flasks were sealed with 45 mm gray bromobutyl rubber anaerobic flanges. Exponential phase cultures (OD_600_ between 0.6 and 1) were immediately cooled on ice, centrifuged at 4°C at 4,000 × *g* for 10 minutes, resuspended in 10 mL of 4°C resuspension buffer (25 mM Tris, 10 mM NaCl, pH 8), centrifuged again at 4°C at 4,000 × *g* for 10 minutes, and decanted. Cell pellets were stored at −80°C for further analysis. Pellets were resuspended in 2 mL of resuspension buffer at 4°C and lysed by a single pass at 25 kPSI through the MC-BA Cell Disruptor (Constant Systems). Lysed cells were centrifuged at 4°C and 14,000 × *g* for 40 minutes in a Multifuge X Pro Series centrifuge (Thermo Scientific). All assays were performed at 30°C in clear flat-bottom polystyrene 96-well plates (Greiner Bio-One). Absorbance, wavelengths indicated below, was monitored every 15 seconds for 5 minutes using a Synergy HTX plate reader (Agilent). A BCA kit (Pierce) was used to determine total protein content by measuring A_562_ in clarified lysates.

### GSH-linked formaldehyde oxidation assay

GSH-linked oxidation assays were performed according to the procedures of Yanpirat et al. (24), except that formaldehyde was prepared by heating and stirring a liquid solution of paraformaldehyde in the fume hood until the solution just began to boil, then immediately sealed. Cell lysate, prepared as indicated above, containing 50 µg–100 µg of total protein was added to each assay, and the production of NADH was monitored via absorbance at 340 nm using an extinction coefficient of 6.22 mM^−1^ to calculate activity (24). Negative controls with no protein added were included and had no change in absorbance over time.

### FDH activity assay

Protocols of Buttery and Chamberlain (46) were used to detect FDH activity by monitoring the reduction of *p*-iodonitrotetrazolium, except that no exogenous FDH was added; instead, cell lysates were used as the source of FDH. Between 50 and 150 µg of total protein were present in each assay, except for the no-protein controls, which exhibited no change in absorbance over time. Cell lysates were incubated at 30°C for 1 minute prior to the assay, and the reaction was initiated as previously described but using 1.67 mM formate. The reaction progress was monitored at 510 nm, and calculations of activity considered an extinction coefficient of 13 mM^−1^ for formazan (47).

### RNA-seq analysis of *Bs.* 3456 grown with methanol + La versus succinate + La

**Fig 2:**
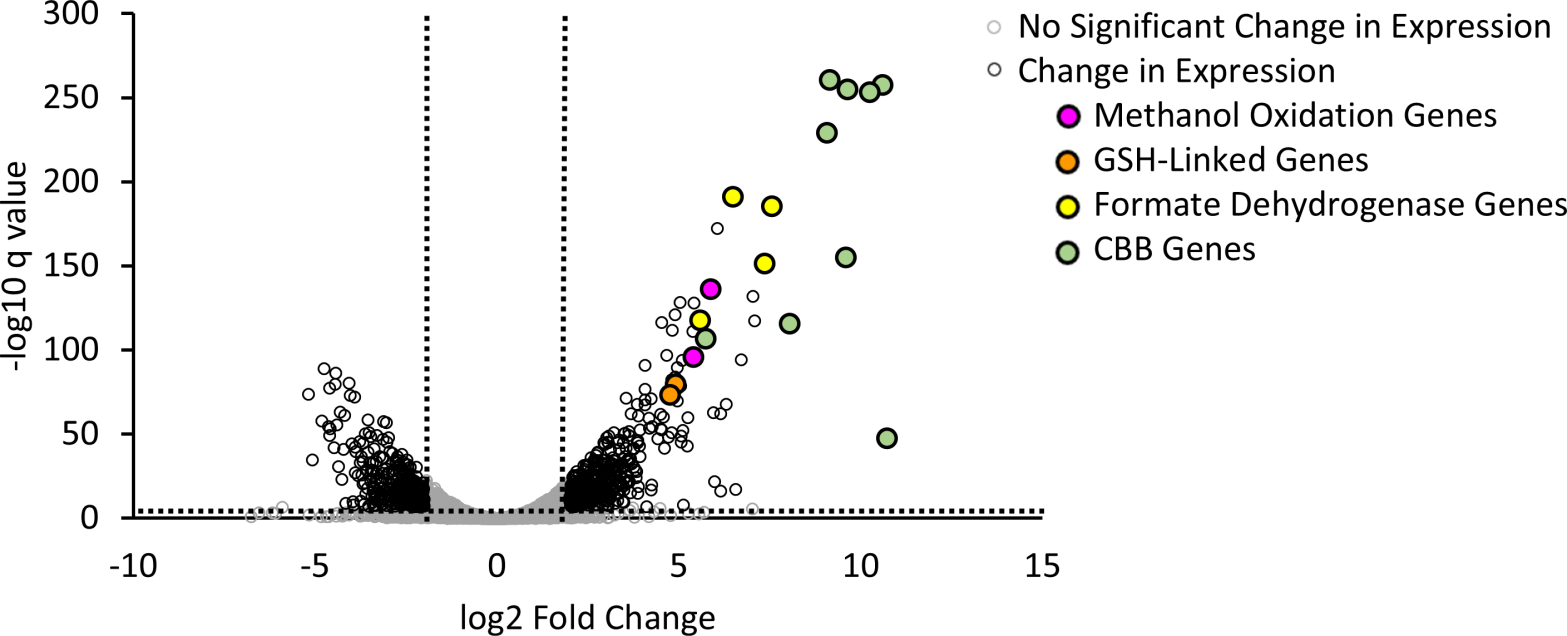
Volcano plot of differentially expressed genes during growth of *Bs*. 3456 with methanol and succinate. Cells were grown with either 15 mM succinate + La (left) or 50 mM methanol + La (right). Transcripts of interest for methanol metabolism are indicated by color-filled points. Genes were classified as differentially expressed using the stringent cut-off of log_2_ fold change greater than 2 and −log_10_ q-value greater than 7 indicated by dashed lines on the volcano plot. Using these parameters, 331 genes were downregulated, and 453 were upregulated out of a total of 10,757 unique genes detected via RNA-seq.

Cultures were grown as previously described, and samples were harvested when cultures reached exponential phase (OD_600_ of 0.3 for methanol and 0.7 for succinate). Total RNA was extracted using the Qiagen RNeasy Kit as described by Okubo et al., except bead beating occurred for only 45 s and SUPERase•In was not needed (48). rRNA was removed via the Ribo-Zero RNA Plus rRNA Depletion Kit (Illumina); library prep and sequencing were performed by SeqCoast Genomics, Portsmouth, NH. Twelve million 2 × 150 bp paired-end reads were generated per sample. Paired-end libraries were uploaded to KBase (49), and reads were aligned to the *Bs*. 3456 genome (downloaded from MaGE accession GCA_006519655.1) with HISAT2 and assembled using StringTie. DeSeq2 was used to analyze expression patterns. Genes were classified as differentially expressed using the stringent cut-off of log_2_ fold change greater than 2 and −log_10_ q-value (*P*-value adjusted for the false discovery rate) greater than 7. Both conditions were analyzed in triplicate to generate Fig. 2 and Table S3. Raw reads and processed data are available from GEO (study number GSE295604), and a table with fragments per kilobase of transcript per million mapped reads (FPKM) values for each sample, q-values, and log_2_ fold change is available at 10.6084 /m9.figshare.30071233.

### SDS-PAGE and 1-D gel digestion proteomics

Clarified cell lysates were mixed with 2× Laemmli buffer + 5% beta-mercaptoethanol and boiled at 95°C for 10 minutes. Twenty micrograms of total protein was present in each sample. Samples were analyzed by gel electrophoresis using a 10-well, 4–20% gradient Mini-PROTEAN TGX Stain-Free Gel run at 150 V with a PageRuler Plus Prestained ladder (Thermo). The gel was stained using Coomassie Blue and imaged using the GelDoc Go, Software Version 3.0.0.07 “Coomassie” program (Bio-Rad). Bands at the predicted molecular weight of RuBisCO (54 kDa) were excised, stored at −20°C, and the protein sequence was identified at the Vincent J. Coates Proteomics Facility at UC Berkeley via reverse-phase liquid chromatography with tandem mass spectrometry (LC-MS/MS).

### Plasmid and strain construction

A plasmid for generating an in-frame markerless deletion of the complete coding region of *cbbS,* which encodes the small subunit of RuBisCO, was constructed in the pCM433KanT backbone which includes kanamycin-resistance genes for selection and *sacB* for counterselection with sucrose (45). Approximately 650 bp of genomic DNA directly upstream and downstream of *cbbS* (AAV28_09890) was amplified with overhangs (Table 1) from the *Bd*. 110 genome and inserted into the pCM433KanT plasmid using HiFi DNA Assembly Master Mix (NEB). The resulting plasmid was sequenced (Plasmidsaurus) and electroporated into *Escherichia coli* S17-1. Biparental mating between *E. coli* S17-1 containing the deletion plasmid and wild-type *Bd*. 110 was used to introduce the plasmid. Mating took place for 48 hours on MP plates containing 1 g/L each of arabinose, gluconic acid, and yeast extract (MP-AGY plates). We determined that *Bd*. 110 is naturally resistant to 5 µg/mL tetracycline when grown on MP-AGY plates, but *E*. coli S17-1 is not, so 5 µg/mL tetracycline and 100 µg/mL kanamycin were used for the first selection and the removal of *E. coli*, followed by a transfer to 5 µg/mL tetracycline and 200 µg/mL kanamycin for a second round of selection, also on MP-AGY plates. Kanamycin-resistant single-crossovers were then transferred to MP-AGY plates supplemented with 10% sucrose, for selection, and 5 µg/mL tetracycline to inhibit the growth of contaminants, including *E. coli*. Resulting colonies were replica patched onto MP-AGY plates supplemented with either 10% sucrose or 200 µg/mL kanamycin. Those that grew with sucrose, but not kanamycin, were PCR screened for the loss of *cbbS* using primers CRM148 and CRM151 (Table 1) and sequenced (Plasmidsaurus) to confirm clean deletion of *cbbS*.

### Liquid chromatography-mass spectrometry to detect ^13^C-labeled intermediates of the CBB cycle

Strains were pre-cultured in MP + 10 µM LaCl_3_ with 50 mM unlabeled methanol as a sole carbon source. Pre-cultures in exponential phase (OD_600_ of 0.5) were centrifuged for 10 minutes at 2,000 × *g*, washed in MP without carbon sources, and transferred to 50 mL of MP media containing 50 mM of either ^13^C-labeled or unlabeled methanol (starting OD_600_ of 0.01). When preparing the cultures, each flask was sealed with gray bromobutyl flanges to promote assimilation of CO_2_ from methanol, instead of unlabeled CO_2_ from the atmosphere. These cultures were grown to exponential phase (OD_600_ of 0.5) and harvested via vacuum filtration on 0.22 µm nylon filters (Micron Separators Inc). Filters were immediately flash-frozen in liquid nitrogen and were stored at −80°C until analysis.

A boiling water extraction was used according to the procedures of Cocuron and Alonso (50), except that tungsten beads were not needed to disrupt the tissues. Samples were flash-frozen, lyophilized, and sent to the University of North Texas BioAnalytical Facility on dry ice. Samples were resuspended and analyzed via reverse-phase anion-exchange LC-MS/MS according to the procedures of (50). Analytes were detected via multiple reaction monitoring, and retention time and fragment masses were compared with external 3-phosphoglycerate (3PGA), fructose 6-phosphate (F6P), glucose 6-phosphate (G6P), ribulose 5-phosphate (R5P), ribulose 1,5-bisphosphate (RuBP), and sedoheptulose 7-phosphate (S7P) standards. The total peak area for each mass isotopomer was calculated and used for comparisons between samples.

### Structural predictions and determination of homology between putative XoxFs

The amino acid sequence of XoxF1 from *Me.* AM1 (META_1740) was queried against the genomes of *Me*. AM1 (chromosome META1.1), *Bd*. 110 (chromosome CP011360.1), *Bs*. 3456 (WGS VIDU01.1), and *Sm.* 2011 (plasmid NC_020560.1, plasmid NC_020527.1, chromosome NC_020528.1) using the BLAST functionality in MaGE, MAgnifying Genomes, with cut-offs of >50% query coverage and >25% identity (51). Twenty hits were returned from among the four genomes. A manual comparison of amino acid sequences determined the presence/absence of an additional aspartate, which was shown to be essential for Ln binding (15). Sequences from the BLAST search were aligned using ClustalW. A phylogenetic tree was constructed using the maximum likelihood method via IQ-Tree with an automatically detected substitution model including free-rate heterogeneity and 1,000 ultrafast bootstrap alignments, and the tree was visualized using iTOL (52–54). All sequences were fed to AlphaFold3 (55) using default parameters, and the overall fold without ligands was assessed. AlphaFold3 models of key PQQ-DH homologs were aligned with the crystal structure of the XoxF1 holoenzyme from *Me*. AM1 (PDB ID: 6OC6) using ChimeraX (15, 56).

### Genomic context of *xoxF* homologs visualized using Clinker

Organisms were selected based on literature reports of relevant 1-carbon metabolism and to provide a diverse array of *Rhizobiales*. Genomic sequences were manually identified based on proximity to *xoxF* homologs and downloaded from the NCBI and aligned via Clinker in CAGECAT (online) with default settings (57, 58).

### Predicting the distribution of the XoxF-CBB pathway among *Rhizobiales* genomes

Select *Rhizobiales* genomes were accessed and analyzed using the IMG database (59). Genomes were searched by BRENDA enzyme commission (EC) numbers and via BLAST with cut-offs of >50% amino acid identity. Abbreviations, EC numbers, and queries are indicated for each enzyme. Methanol oxidation queries were Ca-dependent MDH (MxaF, 1.1.2.7, META1_4538) and Ln-dependent MDH (XoxF, 1.1.2.10, META1_1740) from *Me*. AM1. GSH-linked oxidation pathway queries were S-(hydroxymethyl)glutathione synthase (Gfa, 4.4.1.22, VIDU01_860230), S-(hydroxymethyl)glutathione dehydrogenase (FrmA, 1.1.1.284, VIDU01_860229), and S-formylglutathione hydrolase (FrmB, 3.1.2.12, VIDU01_860197) from *Bs*. 3456. Select genes of the H_4_MPT pathway were formaldehyde-activating enzyme (Fae, 4.2.1.147, META1_1766) and methenyltetrahydromethanopterin cyclohydrolase (Mch, 3.5.4.27, META1_1763) from *Me*. AM1. The canonical H_4_F pathway was queried using formyltetrahydrofolate deformylase (PurU, 3.5.1.10, AGT11408.1) and methenyl tetrahydrofolate cyclohydrolase (FolD, 1.5.1.5, AGT11407.1) from *Paracoccus aminophilus* JCM7686-PMC (60). Formate oxidation capacity was queried using formate dehydrogenase (FDH, 1.17.1.9, 1.17.1.10, 1.2.2.1, META1_5032, META1_4848, META1_2094, and META1_0303) from *Me*. AM1. 3-Hexulose-6-phosphate synthase (Hps, 4.1.2.43, Mfla_0250) and 3-phospho-6-hexuloisomerase (Phi, 5.3.1.27, Mfla_1653) from *Methylobacillus flagellatus* KT were used as markers for the RuMP cycle (61). The glyoxylate shunt was identified by isocitrate lyase (Icl, 4.1.3.1, AAG06022.1) and malate synthase (Ms, 2.3.3.9, AAG03871.1) from *Pseudomonas aeruginosa* PAO1 (62). The CBB was searched using RuBisCO (CbbL, 4.1.1.39, VIDU01_10123) and phosphoribulokinase (Prk, 2.7.1.19, VIDU01_10120) from *Bs*. 3456. The serine cycle was predicted from glycine hydroxymethyltransferase (GlyA, 2.1.2.1, META1_3384), malate thiokinase (MtkA, 6.2.1.9, META1_1730), and malate-CoA lyase (Mcl, 4.1.3.24, META1_1733) from *Me*. AM1. The EMC pathway was indicated by crotonyl-CoA carboxylase/reductase (Ccr, 1.3.1.85, META1_0178) and (2S)-methylsuccinyl-CoA dehydrogenase (Mcd, 1.3.8.12, META1_2223) from *Me*. AM1 (37). The phylogenetic tree was constructed from RpoB amino acid sequences extracted from the IMG database and analyzed using ClustalW and IQ-Tree, with an automatically detected substitution model, and visualized using iTOL (52–54). Bootstrap values are from 1,000 ultrafast calculations in IQ-Tree. The tree was manually annotated to reflect gene presence/absence according to the above analysis.

## RESULTS

### Genomic data suggest the CBB cycle is employed for methanol assimilation

Because multiple methanol assimilation modules have been characterized in the literature, we initially explored genomic data to identify potential pathways employed by strains *Bs*. 3456, *Bd*. 110, and *Sm.* 2011. All three strains lack key genes for both the serine cycle (*mtkA*, *mcl*) and RuMP pathway (*hps*, *phi*) (Tables S1 and S2), and the absence of these genes indicates that the serine and RuMP cycles are incomplete and cannot function in methanol assimilation in *Bs*. 3456, *Bd*. 110, and *Sm.* 2011. Other serine cycle genes, such as *glyA*, *sga*, and *mdh*, and RuMP pathway genes, including *rpi*, *glpx,* and *tkt*, that are present in these strains likely function in overlapping metabolic pathways, such as the citric acid cycle or the pentose phosphate pathway, respectively. However, all three strains possess genes predicted to encode the Ln-dependent dehydrogenase XoxF, the glutathione-linked oxidation pathway, various FDHs, and a complete CBB cycle (Tables S1 and S3). This suite of genes led us to hypothesize that strains *Bs*. 3456, *Bd*. 110, and *Sm.* 2011 can oxidize methanol to CO_2_ to generate energy, enabling them to assimilate either atmospheric or methanol-derived CO_2_ via the CBB cycle (Fig. 1).

The presence of *xoxF* homologs led us to search for appropriate accessory genes for Ln uptake in the organisms of interest. Homologs for genes of the *lut* (lanthanide utilization and transport) cluster from *Me*. AM1, including *lutA*, *B*, *E*, *F*, and *G*, encoding an ABC transport system and periplasmic proteins, can be found in all strains with 40%–60% identity, according to the BLAST function in MaGE (63). Homologs of *lutH*, the TonB-dependent outer membrane transporter, from *Bd*. 110 and *Bs*. 3456 exhibit lower similarity to the sequence from *Me*. AM1 than the other *lut* genes, and no obvious homolog can be found in *Sm.* 2011 using BLAST (Table S3). While it is possible that a *lut* cluster operates in these organisms, it is unlikely that they utilize the small molecule Ln chelator methylolanthanin for Ln solubilization, as none of the strains of interest encode clear homologs of *mllA-E* according to BLAST analysis in the MaGE database (64).

### Transcriptomics reveal upregulation of the proposed methanol assimilation genes

To identify genes likely to be involved in methanol metabolism, RNA-seq was performed on *Bs*. 3456 grown with either 15 mM succinate or 50 mM methanol as sole carbon sources, supplemented with 10 µM La in both conditions. When stringent cut-offs of log_2_ fold change greater than 2 and −log_10_ q-value greater than 7 were applied, 453 genes were identified as upregulated in the methanol condition compared to the succinate condition. Several genes necessary for methanol metabolism were among the most highly upregulated, including the methanol dehydrogenase *xoxF* (VIDU01_860227, 59-fold upregulation) and putative methanol dehydrogenase-associated cytochrome *xoxG* (VIDU01_860228, 43-fold) for methanol oxidation. GSH-linked formaldehyde oxidation genes *gfa* (VIDU01_860230, 30-fold) and *frmA* (VIDU01_860229, 27-fold) were upregulated, as were genes for NAD^+^-dependent formate dehydrogenase subunits alpha, beta, delta, and gamma (VIDU01_10810, 10811, 10808, and 10812, respectively, with 189-, 90-, 166-, and 48-fold upregulation, respectively). Genes for the large and small subunits of RuBisCO, *cbbL* (VIDU01_10123, 1,709-fold) and *cbbS* (VIDU01_10124, 1,563-fold), and other genes of the CBB cycle were upregulated, allowing for CO_2_ fixation (Fig. 2). Genes potentially involved in the serine/EMC cycle or the RuMP pathway are either downregulated or do not change in expression in *Bs*. 3456 during growth with methanol vs succinate (log_2_ fold change of 1.5 or less). This includes serine cycle and EMC pathway genes such as *glyA* (VIDU01_310356), *ppc* (VIDU01_10600), *mdh* (VIDU01_270073), *ccr* (VIDU01_510004), as well as *icl* (VIDU01_320092) and *ms* (VIDU01_640301) from the glyoxylate shunt.

### Identification of XoxFs driving light Ln-dependent methanol oxidation

**Fig 3:**
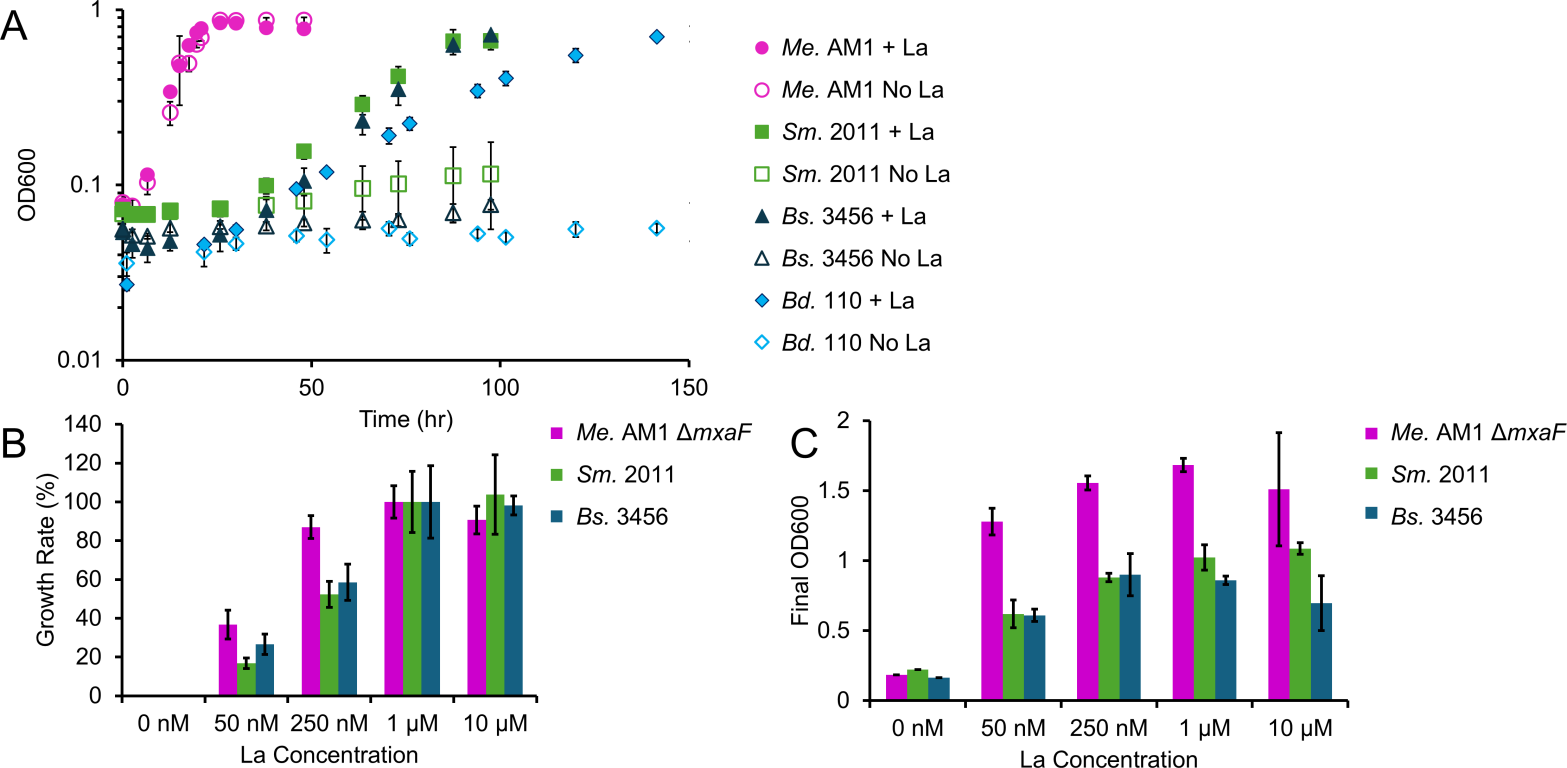
Phenotypic evidence for XoxF as a methanol dehydrogenase involved in Ln-dependent growth with methanol. (**A**) Growth of wild-type *Me*. AM1 (pink circle), *Sm*. 2011, (green squares), *Bs*. 3456 (dark blue triangles), and *Bd*. 110 (light blue diamonds) with 50 mM methanol with (filled) and without (unfilled) 10 µM LaCl_3_. No significant differences in growth rate were found for *Me*. AM1 ± La. Differences were significant ± La for strains *Bs*. 3456, *Bd*. 110, and *Sm.* 2011 (*P* ≤ 0.001 ± La according to a two-tailed *t*-test). Error bars indicate the standard deviation of three replicates. (**B**) Relative growth rates and (**C**) final yields for growth with 50 mM methanol with increased concentrations of LaCl_3_ for *Bs*. 3456, *Sm*.2011, and *Me*. AM1 Δ*mxaF* (relies solely on Ln-dependent PQQ-DHs). Error bars indicate the standard deviation of three replicates. According to a two-tailed Student’s *t*-test, all strains exhibit a statistically significant (*P* ≤ 0.05) difference in growth rate across all lanthanum concentrations, except for the 1 vs 10 µM comparison, and all strains exhibit a statistically significant (*P* ≤ 0.05) difference in final OD across all lanthanum concentration comparisons, except for 50 nM vs 10 µM, 250 nM vs 1 µM, and 250 nM vs 10 µM for *Bs*. 3456, 250 nM vs 1 µM and 1 µM vs 10 µM for *Sm*. 2011, and 5 nM vs 10 µM, 250 nM vs 1 µM, and 1 µM vs 10 µM for *Me*. AM1.

The ability to assimilate methanol was expected for *Bd*. 110 due to prior reports in the literature (13). Here, we show that *Bd*. 110, *Bs*. 3456, and *Sm*. 2011 grow with methanol only when light Lns are present, unlike wild-type *Me*. AM1, which encodes both *mxaF* and *xoxF* and grows in the presence and absence of Lns (Fig. 3A; Fig. S1). Higher concentrations of Lns support faster growth rates and higher final yields, with maxima observed at 1 μM–10 μM La (Fig. 3). In addition to the presence of Lns, *Bd*. 110 required sealed Balch-style tubes for growth with methanol (Fig. S2), but further increasing the CO_2_ levels to 0.08, 0.2, or 5% (vol/vol) in the headspace did not further increase growth rate or final yield (data not shown).

Potential XoxF homologs from *Bs*. 3456, *Bd*. 110, and *Sm*. 2011 were compared to the crystal structure of XoxF1 from *Me*. AM1 (PDB structure 6OC6). *Me*. AM1 encodes three different Ln-dependent dehydrogenases, but XoxF1 is the principal PQQ-MDH for methanol oxidation (15). Similarly, *Bd*. 110, *Bs*. 3456, and *Sm*. 2011 each encode multiple predicted Ln-dependent PQQ dehydrogenases (Fig. S3). We identified one homolog from each organism that has the highest sequence similarity to XoxF1 and XoxF2 from *Me* AM1. Notably, for *Bs*. 3456, the closest XoxF homolog (VIDU01_860227) is also the most highly upregulated PQQ dehydrogenase transcript (60-fold upregulation) during growth with methanol and La compared to succinate and La (Fig. 2). All XoxF homologs were 601–602 amino acids in length and shared approximately 75% identity with XoxF1 and XoxF2 from *Me*. AM1. When modeled in AlphaFold 3, all structures were predicted with >90% confidence, except for an approximately 25 amino acid sequence at the N-terminus that is predicted to be a signaling peptide that promotes trafficking to the periplasmic space, according to InterPro (65). The presence and orientation of residues in the active site is particularly important for substrate specificity and for the ability to bind Ln^3+^. A model of VIDU01_860227 is shown compared to XoxF1 from *Me*. AM1, and a magnified view highlights the conserved orientation of the predicted catalytic aspartate and the Ln-binding aspartate (pink) (Fig. S3). The putative XoxFs from *Bd*. 110 and *Sm*. 2011 adopt a similar overall fold and active site conformation.

### GSH-linked genes are adjacent to *xoxF*, and the GSH-linked pathway for formaldehyde oxidation is active during growth with methanol

**Fig 4:**
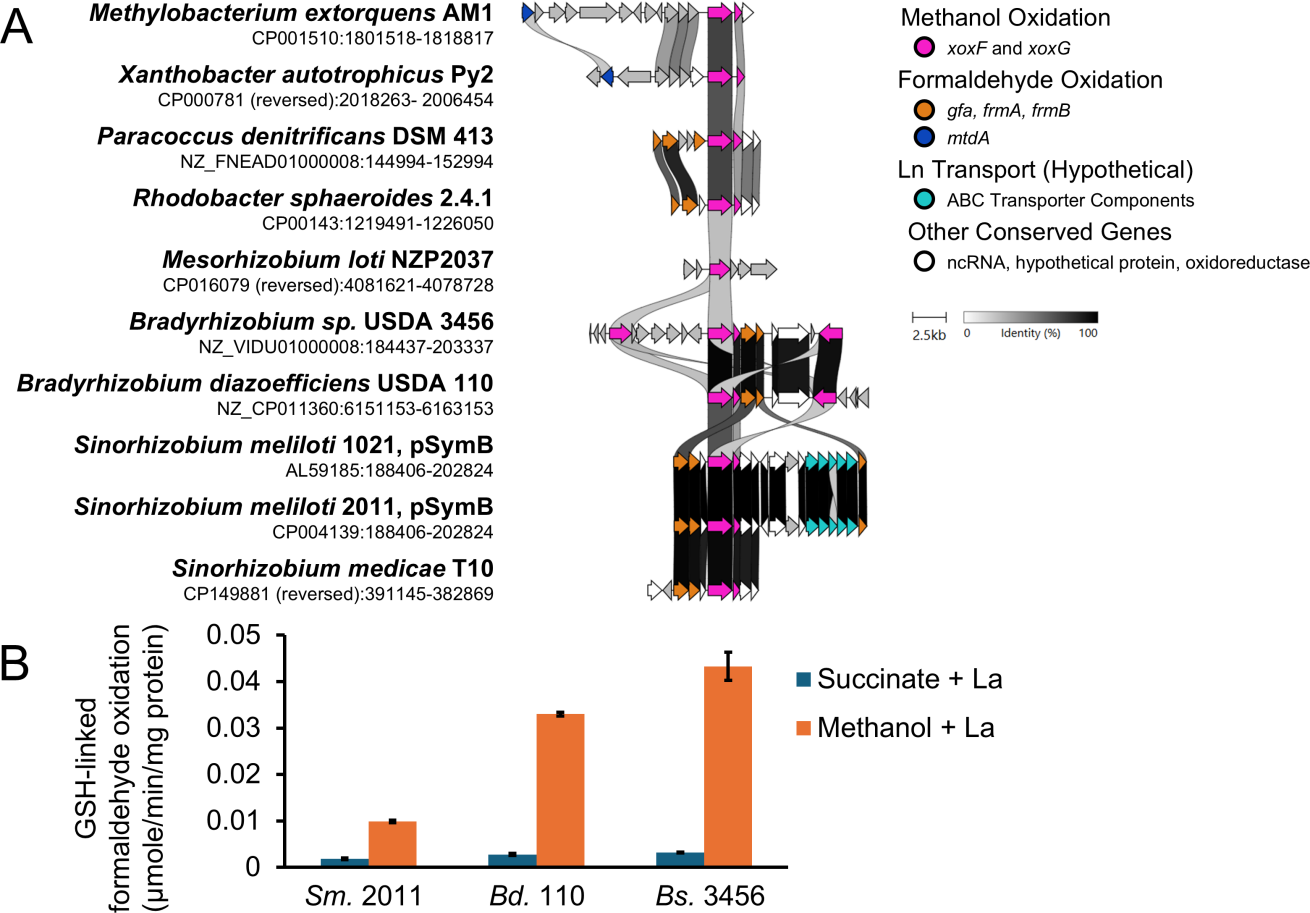
GSH-linked pathway implicated in methanol metabolism. (**A**) Visualization of the region surrounding *xoxF* homologs in select Alphaproteobacteria generated using Clinker on the CAGECAT web server (57, 58). Arrows represent genes. Colored arrows represent genes implicated in methanol metabolism with greater than 30% sequence similarity with homologs from other selected strains. Genes represented by white arrows have homologs in the data set, but no known role in methanol metabolism. Genes in gray have less than 30% similarity to other genes in this data set. Homologous genes are connected by ribbons, and darker ribbon color indicates higher similarity. Note that *Bs*. 3456 encodes three *xoxF* homologs in the selected region (VIDU01_860219, 860227, and 860235), and *Bs*. 110 encodes two *xoxF* homologs (AAV28_28625 and 28655) in the selected region. Genome accession numbers and coordinates are indicated below each strain name. (**B**) NADH-linked activity assay for glutathione-dependent formaldehyde dehydrogenase activity. All strains exhibit a statistically significant (*P* ≤ 0.001) difference in activity between succinate- (blue) and methanol-grown (orange) cultures according to a two-tailed Student’s *t*-test. Error bars indicate the standard deviation of three replicates.

Based on genomic evidence from all strains and transcriptomic evidence from *Bs*. 3456, we predicted that the GSH-linked oxidation pathway was used to oxidize formaldehyde to formate during growth with methanol (Fig. 2; Table S2). In addition, visualizing the genomic content of GSH-linked oxidation pathway components (*gfa*, *frmA*, *frmB*) indicates that these genes cluster with *xoxF* homologs in *Sm*. 2011, *Bd*. 110, and *Bs*. 3456 (Fig. 4A). A similar organization is observed in select *Paracoccus denitrificans* and *Rhodobacter sphaeroides,* and *Sinorhizobium medicae* strains. In organisms that do not use the GSH-linked oxidation pathway, like *Me*. AM1 and *Xanthobacter autotrophicus*, *mtdA* and *fch* are involved in formaldehyde metabolism and are adjacent to *xoxF* homologs in their respective organisms. This conserved organization suggests an evolutionary pressure to co-regulate these genes across divergent organisms and pathways.

We used the GSH-linked oxidation assay, which monitors NADH produced by the GSH-dependent formaldehyde dehydrogenase, FrmA, to demonstrate that the GSH-dependent formaldehyde oxidation pathway is functional during growth with methanol. Lysates from *Sm.* 2011, *Bs*. 3456, and *Bd*. 110 cells grown with methanol have 5, 11, and 13 times greater GSH-dependent formaldehyde oxidation activity, respectively, compared to lysates from cells grown with succinate (Fig. 4B). Notably, activity from *Sm.* 2011 extracts grown with methanol is only 25% of the activity observed with *Bs*. 3456 extracts grown with methanol.

### NAD^+^-dependent FDH activity is elevated during growth with methanol

**Fig 5:**
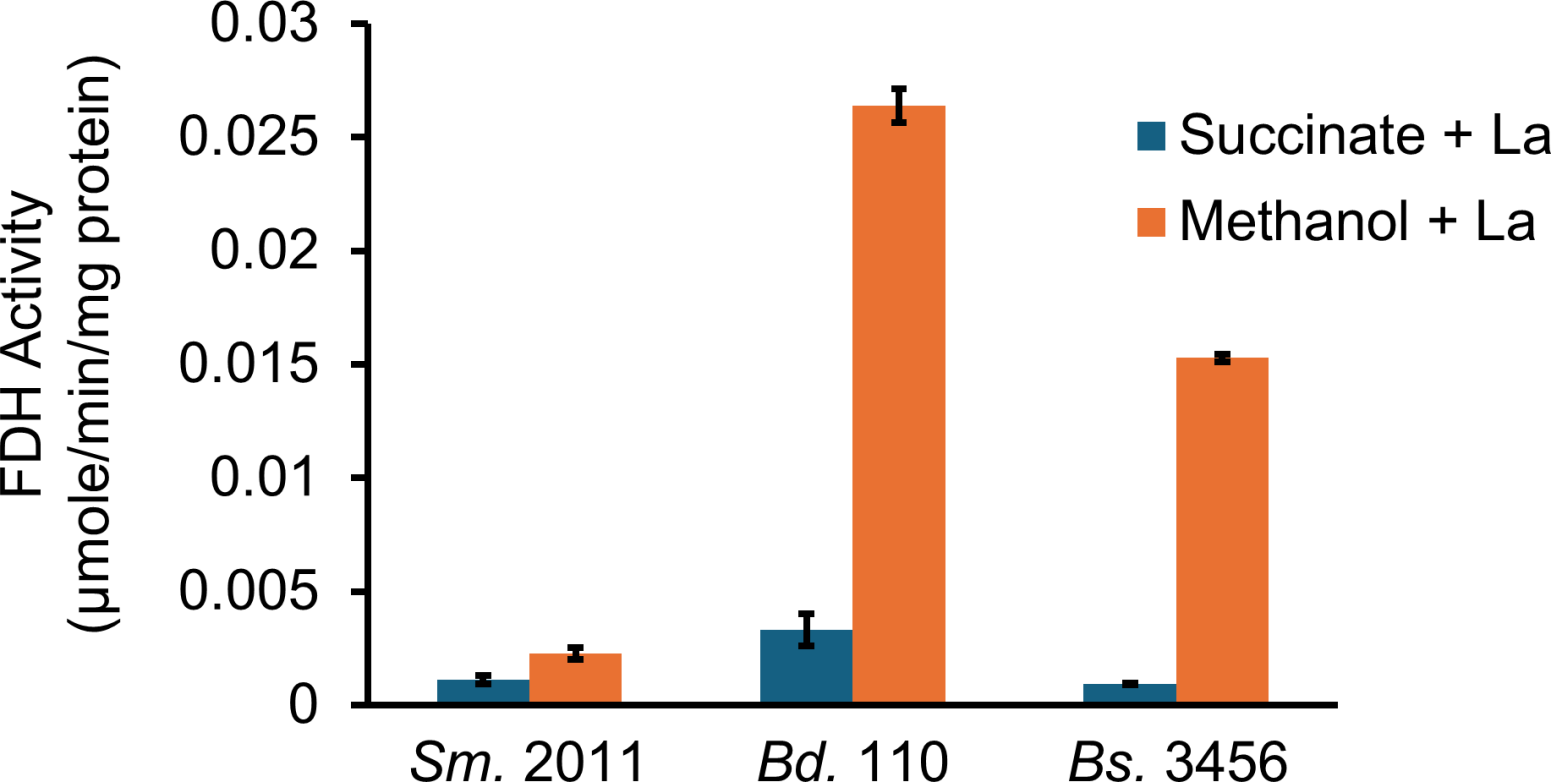
NAD^+^-dependent formate dehydrogenase activity assay. All strains exhibit a statistically significant (*P* ≤ 0.005) difference in activity between (blue) and methanol (orange) grown cultures according to a two-tailed Student’s *t*-test. Error bars indicate the standard deviation of three replicates.

FDH activity is needed to generate NADH and produce CO_2_ for carbon assimilation via the CBB cycle. To determine whether apparent FDH activity was greater in cells grown with methanol versus succinate, we employed a formate dehydrogenase activity assay to quantify the ability of cell lysates to convert formate to CO_2_. *Me*. AM1 encodes four FDHs with redundant activity (26), and one or more homologs of each FDH type is found in the *Bd*. 110 and *Bs*. 3456 genomes (Table S3). In contrast, a single NAD^+^-dependent homolog of Fdh2A was identified in *Sm.* 2011 (Table S3). As expected, for all strains, there was greater FDH activity in lysates from cells grown with methanol than those grown with succinate (Fig. 5). Activity in methanol-grown cultures, compared to succinate-grown cultures, was eightfold greater for *Bd*. 110 and 16-fold greater for *Bs*. 3456. FDH activity observed from *Sm.* 2011 lysates grown with methanol was less than 10% of that observed for *Bs*. 3456, but activity from *Sm.* 2011 cultures was twofold higher in methanol-grown cultures than succinate-grown cultures.

### The CBB cycle is used for assimilation during growth with methanol

**Fig 6:**
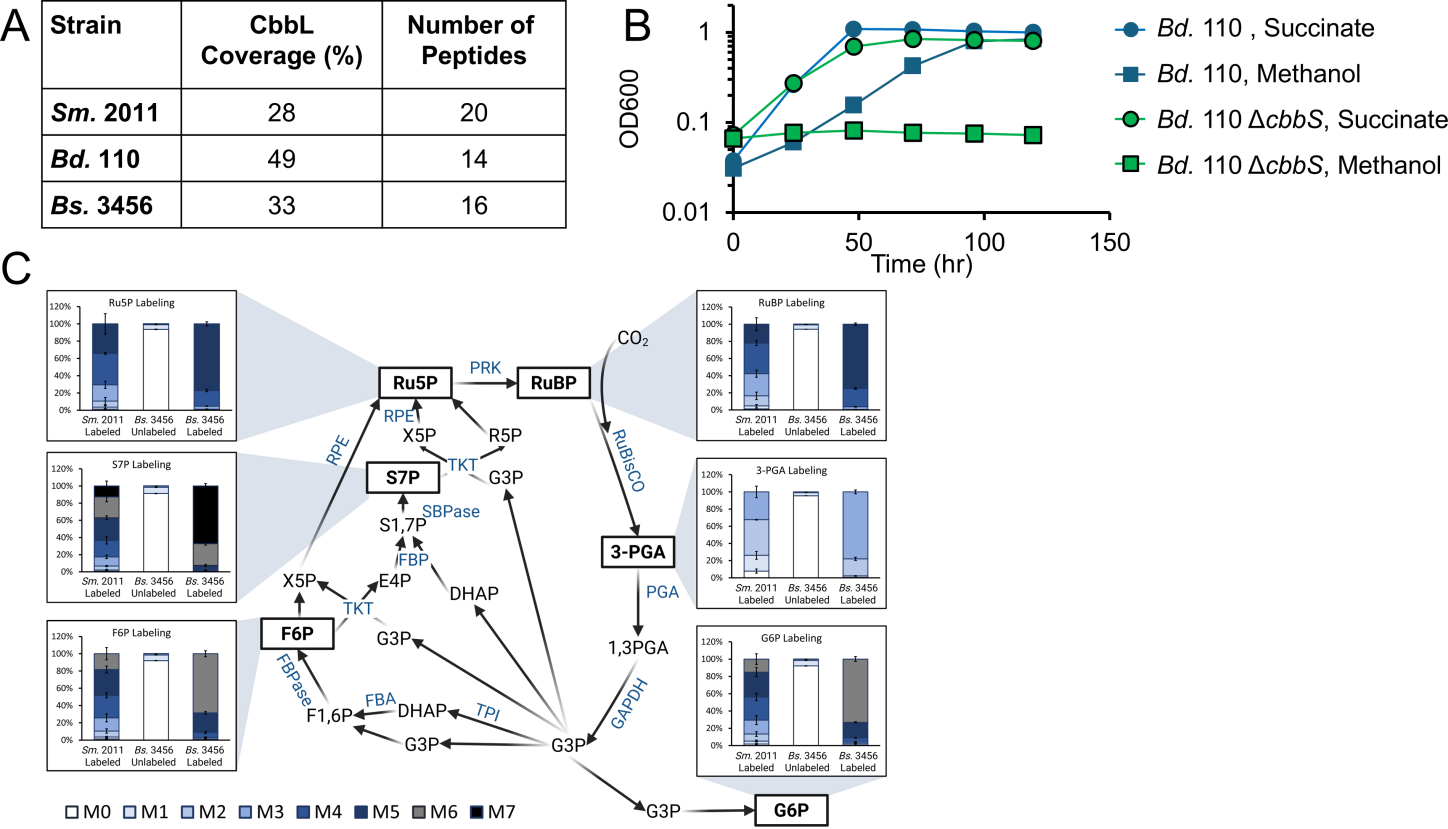
Involvement of the CBB cycle in methanol metabolism. (**A**) Coverage and number of peptides from RuBisCO detected in methanol-grown lysates of *Bd*. 110, *Bs*. 3456, and *Sm.* 2011 via gel digestion LC-MS. (**B**) Growth of wild-type (blue) and Δ*cbbS Bs*. 110 (green) with 15 mM succinate + 10 µM La (circles) and 50 mM methanol + 10 µM La (squares) in sealed Balch-style tubes. Error bars indicate the standard deviation of three replicates. (**C**) The percentage of each mass isotopomer for six different analytes is depicted for labeled *Sm.* 2011, unlabeled *Bs*. 3456*,* and labeled *Bs*. 3456. Error bars indicate the standard deviation of three replicates. The isotopomer color-coding key at the bottom holds true for all analytes. Metabolites are spatially organized according to their role in the canonical CBB cycle, and relative isotopomer abundance is shown near each measured metabolite. Data are the result of three biological replicates analyzed via reverse-phase anion-exchange LC-MS utilizing multiple reaction monitoring. Metabolites are given in black, and enzymes are shown in blue. Metabolites with a box around them have previously been detected via this method and were analyzed in this experiment.

Formate oxidation by FDHs generates CO_2_, which we hypothesized is assimilated via the CBB cycle. Our transcriptomics from *Bs*. 3456 indicate that the CBB cycle is upregulated during growth with methanol compared to with succinate, with approximately 1,600-fold upregulation of *cbbL* and *cbbS*, and 540-fold and 770-fold upregulation of *rpe* and *prk*, respectively (Fig. 2). To validate a functional CBB cycle, the presence of the large subunit of RuBisCO (CbbL) was confirmed in all our organisms of interest during growth with methanol via mass spectrometry by analyzing bands at approximately 55 kDa from all samples grown with methanol (Fig. 6; Fig. S4). A type IC RuBisCO was detected in all of these samples with 28%–49% coverage of the CbbL sequence. Unlike the *Bradyrhizobium*, *Sm*. 2011 encodes both a type IC RuBisCO (SM2011_b20198) and a type II RuBisCO (SM2011_b20393), characterized by lower specificity for CO_2_ over O₂ compared to type IC (66), but only the type IC was detected in this data set.

The essentiality of RuBisCO, and by extension the CBB cycle, for growth of *Bd*. 110 with methanol was also confirmed genetically by constructing an in-frame markerless deletion of the complete coding sequence for the small subunit of RuBisCO (*cbbS*) in *Bd*. 110. *Bd*. 110 Δ*cbbS* was unable to grow with methanol across three biological replicates (Fig. 6B). *Bd*. 110 Δ*cbbS* was still able to grow with succinate at levels similar to wild type, indicating that lack of growth with methanol is due to the assimilatory role of RuBisCO, and not due to non-specific deficits of this strain. Due to challenges with molecular cloning in strains *Bs*. 3456 and *Sm.* 2011, deletions of *cbbS* could not be generated and alternative approaches were employed to provide evidence for operation of the CBB cycle in these strains.

*Bs*. 3456 and *Sm.* 2011 were fed ^13^C-methanol, and incorporation of labeled carbon into CBB cycle intermediates was used for confirmation of CBB cycle activity. All flasks were sealed during growth with ^13^C-methanol to prevent the escape of ^13^CO_2_, and cultures of *Bs*. 3456 and *Sm.* 2011 fed with ^13^C-methanol underwent multiple doublings, 2.6 for *Sm.* 2011 and 6.2 for *Bs*. 3456, leading to a high degree of ^13^C labeling in the analytes of interest. All CBB cycle metabolites of interest (R5P, RuBP, 3PGA, G6P, F6P, and S7P) were successfully detected and labeled in cultures of *Bs*. 3456 and *Sm.* 2011 fed ^13^C-methanol (Fig. 6C). For *Bs*. 3456 and *Sm.* 2011 samples, less than 2% of the M0 mass isotopomer remained for all analytes, indicating a high degree of label incorporation. For *Bs*. 3456, the heaviest possible isotopomer was the most prevalent for each analyte, whereas the second heaviest was most prevalent for *Sm.* 2011, consistent with fewer doublings from *Sm.* 2011. Unlabeled controls from *Bs*. 3456 cultures fed ^12^C-methanol exhibited labeling patterns consistent with the isotopic distribution of unenriched carbon and with the observed isotopomer distributions of the unlabeled external standards.

### Genomic analyses suggest that the capacity for XoxF-CBB metabolism is prevalent among *Bradyrhizobium, Mesorhizobium,* and *Sinorhizobium*

**Fig 7:**
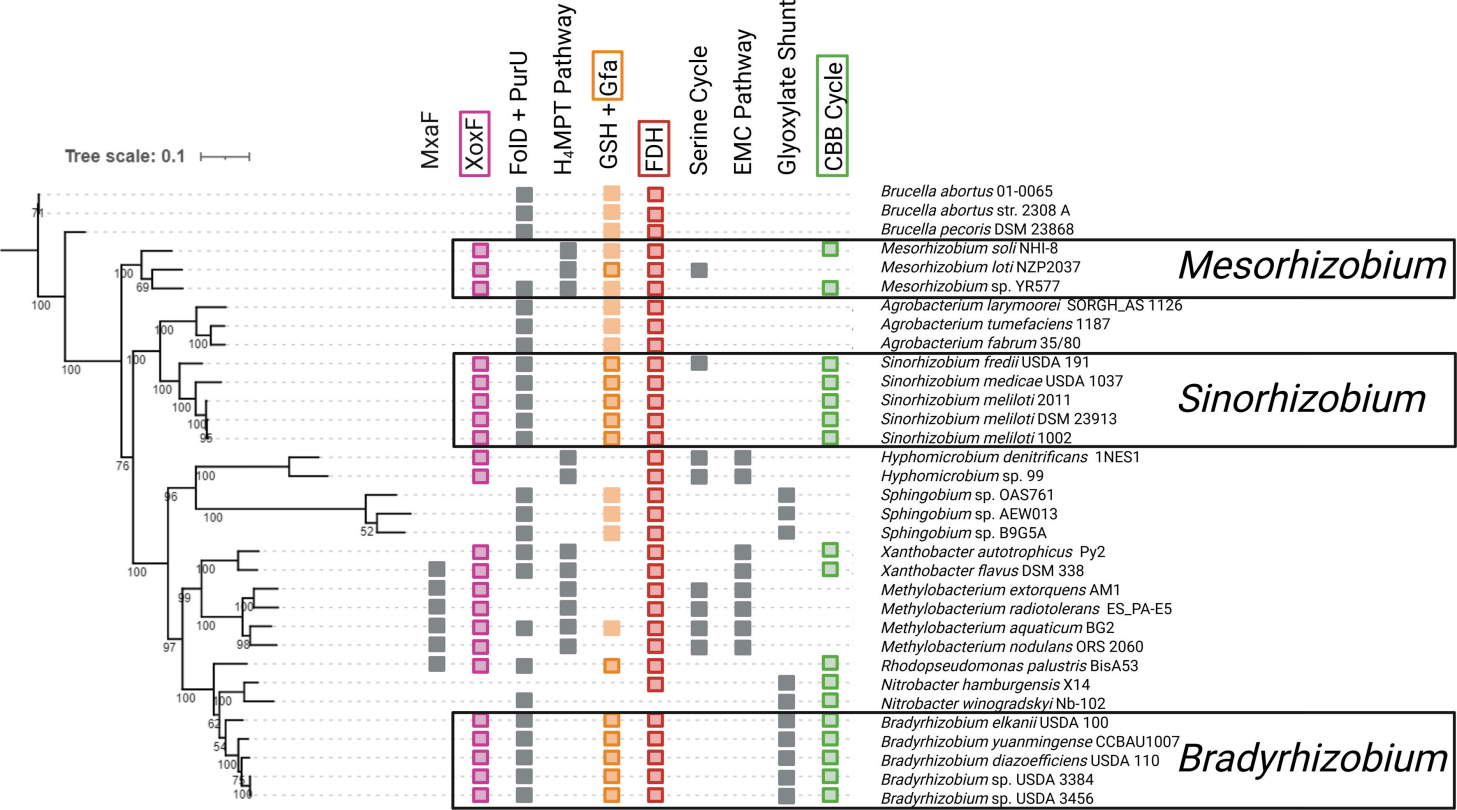
Genomic analyses suggest that the XoxF-CBB pathway for methanol metabolism is widespread among the *Rhizobiales*. Representative *Rhizobiales* were randomly selected from the IMG database, and the phylogenetic tree was constructed using amino acid sequences of RpoB with bootstrap values given at each node. Squares represent the presence of select genes and pathways; no square indicates that no homologs could be found with the given EC number annotated or with >50% sequence identity with the queries indicated in the methods. Steps of the XoxF-CBB pathway are indicated using colored squares, and gray squares are used for other potential methanol assimilation pathways. For the GSH-linked oxidation pathway, plain orange boxes indicate that only *frmA* and *frmB* homologs could be found, while those with a dark orange border also encode *gfa* homologs. None of the selected strains encode both *phi* and *hps* to operate the RuMP pathway. Created in https://BioRender.com.

To address whether the XoxF-CBB pathway is widespread within Alphaproteobacteria, diverse members of the *Rhizobiales* clade were queried for the presence of methanol assimilation genes. As a control, genomes of *Methylobacterium* strains were analyzed using the same methodology and the four *Methylobacterium* strains tested encode genes of the H_4_MPT pathway, serine cycle, and EMC pathway. All tested members of the *Mesorhizobium*, *Sinorhizobium*, and *Bradyrhizobium* clades lack key genes for the complete serine/EMC pathway. None of the selected strains encode a homolog of hexulose-6 phosphate synthase, and only *Mesorhizobium* sp. NZP2037 encodes a copy of 6-phospho 3-hexuloisomerase for operation of the RuMP pathway (data not shown). However, many of these strains are predicted to encode genes for XoxF, the GSH-linked pathway, FDHs, and the CBB cycle (Fig. 7).

## DISCUSSION

Lns are being introduced, both intentionally and inadvertently, at variable concentrations into agricultural soils via commercial fertilizers, making it important to understand how these metals might influence the growth of microbes and, consequently, the productivity of crops (67). The role of Lns in methanol metabolism of the model methylotroph *M. extorquens* has been extensively characterized *in vitro*, and *in planta* studies have determined that *xoxF* is expressed in the plant phyllosphere and confers competitive advantages (9, 15, 64, 68, 69). The effects of Lns on *Rhizobiales*-plant interactions have not yet been characterized, but methanol metabolism of *Methylobacterium nodulans* has been shown to increase the growth of *Crotalaria podocarpa*, raising the potential for similar beneficial effects with other nodule-forming bacteria (70). Lns have been shown to enable growth with methanol in *Bradyrhizobium*, but the pathway enabling growth with methanol and the extent of this metabolism among nitrogen-fixing *Rhizobiales* has yet to be determined (12, 13), despite their essential roles as rhizosphere plant symbionts. Here, transcriptomic, biochemical, metabolomic, and bioinformatic evidence demonstrates that the characterized *Bradyrhizobium* and *Sinorhizobium* strains oxidize methanol using the Ln-dependent PQQ-MDH XoxF, oxidize formaldehyde using the GSH-linked oxidation pathway, oxidize formate via FDHs, and assimilate carbon as CO_2_ using the CBB cycle.

Evidence for the complete XoxF-CBB pathway is provided by transcriptomics of *Bs*. 3456 in which all of the modules involved in methanol assimilation were upregulated during growth with methanol compared to succinate (Fig. 2). Because La was present in both conditions, there was no upregulation of the predicted Ln transport and uptake cluster. Similarly, the lack of upregulation of the PQQ biosynthesis genes suggests that PQQ is synthesized regardless of carbon source, or that the presence of Lns leads to the expression of PQQ biosynthesis genes in both conditions. *xoxF1* (VIDU01_860227) is predicted to encode an Ln-dependent PQQ-DH with 76% similarity to XoxF1 from *Me*. AM1 and is the most highly upregulated PQQ-dependent dehydrogenase, suggesting either a key role in methanol metabolism or regulation by substrate availability. Consistent with the upregulation of a *xoxF*1 homolog, growth with methanol is completely dependent on the presence of Ln metals for wild-type *Bs*. 3456 (Fig. 3A). Additionally, growth rate and final yield are “tunable” and increase as the amount of La in the media increases up to a maximum at 1 μM–10 μM (Fig. 3B and C). Seven other PQQ dehydrogenases are encoded by *Bs*. 3456 with lower similarity to XoxF1 from *Me*. AM1, but their functions are currently unknown (Fig. S3). Assays for S-(hydroxymethyl)glutathione dehydrogenase (FrmA) and FDH activity, together with ^13^C labeling, confirm that the GSH-linked oxidation pathway, FDHs, and CBB cycle are functional during growth with methanol, consistent with the transcriptomic data. Detection of labeled intermediates from cultures supplied with ^13^C-methanol further indicates that *Bs*. 3456 and *Sm.* 2011 are capable of assimilating methanol-derived ^13^CO_2_ and are not reliant on atmospheric ^12^CO_2_ (Fig. 6).

In our hands, *Bd*. 110 was unable to grow with methanol and La unless cultured in sealed Balch-style tubes (Fig. S3). Our initial attempts to grow *Bd*. 110 with methanol and La were unsuccessful, and we hypothesized that *Bd*. 110 could be sensitive to oxygen due to the poor selectivity of RuBisCO for CO_2_ over O_2_. We sealed Balch-style tubes, such that the microbes have a finite amount of oxygen available, and compared growth in this sealed condition to growth in unsealed tubes where diffusion maintains atmospheric oxygen levels. Oxygen cannot be fully removed due to the requirement for a terminal electron acceptor for methanol oxidation in aerobically respiring methylotrophs. *Bs*. 3456 exhibited a shorter lag in sealed tubes compared to foil-covered tubes, but *Bd*. 110 was only able to grow in the sealed tubes (Fig S2). *Me*. AM1, which does not use the CBB cycle, exhibits a slightly reduced growth rate in sealed tubes compared to foil-covered tubes. While other O_2_- or CO_2_-sensitive enzymes besides RuBisCO could be responsible for these results, the striking requirement for sealed conditions for the growth of *Bd*. 110 with methanol indicates that screens for Ln-dependent methylotrophs need to incorporate a low-oxygen condition, and this may be especially important for organisms predicted to use the CBB cycle.

RuBisCO has been shown to be important in multiple plant-*Bradyrhizobium* interactions, though the exact mechanisms for this effect remain unclear. A photosynthetic strain, *Bradyrhizobium* sp. ORS278, in symbiosis with *Aeschynomene indica,* exhibits a defect in symbiotic nitrogen fixation but no defect in free-living nitrogen fixation when the large subunit of RuBisCO (*cbbL*) is disrupted (71). Notably for our study, when the *cbbL* sequence in *Bradyrhizobium diazoefficiens* USDA 110 (*Bd*. 110 in our shorthand) is disrupted, the mutants are less able to colonize roots and occupy nodules of soybean, suggesting that RuBisCO is involved in establishing plant-microbe symbioses (72). The role of RuBisCO in methanol metabolism for these *Bradyrhizobium* strains may contribute to these phenotypes, but further work is needed to confirm this hypothesis.

To our knowledge, the Ln-dependent growth with methanol observed here (Fig. 3) is the first report of Ln-dependent metabolism among the *Sinorhizobium*. Growth with methanol requires nanomolar to micromolar concentrations of La, and only light lanthanides facilitate the growth of *Sm.* 2011 with methanol (Fig. S1). In *Sm.* 2011, genes for XoxF, the GSH-linked pathway, Ln uptake, the CBB cycle, and PQQ biosynthesis are found adjacent to one another in a 28.5 kb region on the symbiotic megaplasmid pSymB. Proteomic analysis of gel-digested bands indicated that *Sm.* 2011 expressed the type IC *cbbL* found in this cluster, instead of the type II *cbbL* that is encoded further downstream on pSymB. Having nearly the complete suite of genes implicated in XoxF-CBB metabolism found on a megaplasmid increases the likelihood of horizontal gene transfer for methanol-related genes among the *Sinorhizobium* (73).

Like the *Bradyrhizobium*, *Sm*. 2011 cultures grown with methanol exhibit greater GSH-linked oxidation activity than those grown with succinate. However, *Sm*. 2011 demonstrates lower GSH-linked oxidation activity than the *Bradyrhizobium* (Fig. 4B). This may result from slight differences in the enzymes, leading to variations in turnover or sensitivity to assay conditions. FDH activity was also lower from *Sm.* 2011 than the *Bradyrhizobium* (Fig. 5). Sm2011_c04444 is the sole FdhA homolog found in *Sm.* 2011 and has 75% sequence similarity to Fdh*2*A from *Me*. AM1 and less than 40% similarity to other FDHs. Fdh2A from *Me*. AM1 is six times less active in *in vitro* FDH assays compared to other FDHs present in *Me*. AM1 (26), explaining the low FDH activity from *Sm.* 2011. The *Bradyrhizobium* encodes homologs of FDHs 1–4, consistent with their higher apparent FDH activity compared to *Sm.* 2011.

The proposed methanol assimilation pathway for *Sm.* 2011, *Bd*. 110, and *Bs*. 3456 is unlikely to be limited to these organisms alone. Instead, the XoxF-CBB pathway appears widespread among the *Bradyrhizobium*, *Sinorhizobium*, and *Mesorhizobium*. Selected members of these genera lack a complete serine/EMC cycle or RuMP pathway, but several encode a *xoxF* homolog as well as genes of the GSH-linked oxidation pathway and the CBB cycle (Fig. 7). This distribution implies that the capacity for Ln-dependent methanol assimilation using the CBB cycle may be more common in the rhizosphere than previously recognized. More work is needed to determine the conditions under which the XoxF-CBB pathway functions in the environment and how this pathway impacts microbial metabolism and influences plant health.

## Data Availability

Data are available from the corresponding author upon reasonable request. Transcriptomic data can be accessed via the GEO database (study number GSE295604) at https://www.ncbi.nlm.nih.gov/geo/query/acc.cgi?acc=GSE295604.

## SUPPLEMENTAL MATERIAL

Supplemental material aem.01304-25-s0001.docx Tables S1 to S3 and Fig. S1 to S4.

ASM does not own the copyrights to Supplemental Material that may be linked to, or accessed through, an article. The authors have granted ASM a non-exclusive, world-wide license to publish the Supplemental Material files. Please contact the corresponding author directly for reuse.
