# Supplementary material for: XoxF and the Calvin-Benson cycle mediate lanthanide-dependent growth on methanol in Bradyrhizobium and Sinorhizobium
Source: Appl Environ Microbiol. 2025 Oct 21;91(11):e01304-25. doi: 10.1128/aem.01304-25 (PMC12628762; doi:10.1128/aem.01304-25)
Supplement: Supplemental material — Tables S1 to S3 and Fig. S1 to S4. [file aem.01304-25-s0001.docx]

**Supplementary Information**

Table S1. Accession numbers from MaGE and NCBI for *Bs.* 3456 genes of interest.

| **MaGE Accession** | **NCBI Locus Tag** | **NCBI Protein ID** | **Gene Name** |
| --- | --- | --- | --- |
| [VIDU01_10119](https://mage.genoscope.cns.fr/microscope/mage/getInfoLabel.php?id=76482486) | FKP26_RS00560 | [WP_027558225.1](https://www.ncbi.nlm.nih.gov/protein/WP_027558225.1?report=genbank&log$=prottop&blast_rank=1&RID=9R8SM3ZD013) | *gplx* |
| VIDU01_10120 | FKP26_RS00565 | [WP_027558226.1](https://www.ncbi.nlm.nih.gov/protein/WP_027558226.1?report=genbank&log$=prottop&blast_rank=1&RID=9R8UX004013) | *prk* |
| [VIDU01_10121](https://mage.genoscope.cns.fr/microscope/mage/getInfoLabel.php?id=76482488) | FKP26_RS00570 | [WP_027558227.1](https://www.ncbi.nlm.nih.gov/protein/WP_027558227.1?report=genbank&log$=prottop&blast_rank=1&RID=9R8WM8UB013) | *tkt* |
| VIDU01_10123 | FKP26_RS00580 | [WP_027558229.1](https://www.ncbi.nlm.nih.gov/protein/WP_027558229.1?report=genbank&log$=prottop&blast_rank=1&RID=9R8YNYXX013) | *cbbL* |
| VIDU01_10124 | FKP26_RS00585 | [WP_027558230.1](https://www.ncbi.nlm.nih.gov/protein/WP_027558230.1?report=genbank&log$=prottop&blast_rank=1&RID=9R90NV4H013) | *cbbS* |
| VIDU01_10125 | FKP26_RS00590 | [WP_027558231.1](https://www.ncbi.nlm.nih.gov/protein/WP_027558230.1?report=genbank&log$=prottop&blast_rank=1&RID=9R90NV4H013) | *cbbX* |
| [VIDU01_10600](https://mage.genoscope.cns.fr/microscope/mage/getInfoLabel.php?id=76482967) | FKP26_RS02800 | [WP_027558646.1](https://www.ncbi.nlm.nih.gov/protein/WP_027558646.1?report=genbank&log$=prottop&blast_rank=1&RID=9R96ANDA013) | *ppc* |
| [VIDU01_10717](https://mage.genoscope.cns.fr/microscope/mage/getInfoLabel.php?id=76483084) | FKP26_RS03340 | [WP_027558747.1](https://www.ncbi.nlm.nih.gov/protein/WP_027558747.1?report=genbank&log$=prottop&blast_rank=1&RID=9R97XVH6015) | *mcmA* |
| VIDU01_10808 | FKP26_RS03760 | [WP_027558825.1](https://www.ncbi.nlm.nih.gov/protein/WP_027558825.1?report=genbank&log$=prottop&blast_rank=1&RID=9R9G51X7015) | *fdh2D* |
| VIDU01_10810 | FKP26_RS03770 | [WP_027558827.1](https://www.ncbi.nlm.nih.gov/protein/WP_027558825.1?report=genbank&log$=prottop&blast_rank=1&RID=9R9G51X7015) | *fdh2A* |
| VIDU01_10811 | FKP26_RS03775 | [WP_027558828.1](https://www.ncbi.nlm.nih.gov/protein/WP_027558825.1?report=genbank&log$=prottop&blast_rank=1&RID=9R9G51X7015) | *fdh2B* |
| VIDU01_10812 | FKP26_RS03780 | [WP_027558829.1](https://www.ncbi.nlm.nih.gov/protein/WP_027558825.1?report=genbank&log$=prottop&blast_rank=1&RID=9R9G51X7015) | *fdh2G* |
| [VIDU01_11139](https://mage.genoscope.cns.fr/microscope/mage/getInfoLabel.php?id=76483506) | FKP26_RS05325 | [WP_027559119.1](https://www.ncbi.nlm.nih.gov/protein/WP_027559119.1?report=genbank&log$=prottop&blast_rank=1&RID=9R9JW1YD015) | *fdh4A* |
| [VIDU01_110101](https://mage.genoscope.cns.fr/microscope/mage/getInfoLabel.php?id=76483983) | FKP26_RS27355 | [WP_027562215.1](https://www.ncbi.nlm.nih.gov/protein/WP_027562215.1?report=genbank&log$=prottop&blast_rank=1&RID=9R9PJ94B015) | *fdh1A* |
| [VIDU01_120226](https://mage.genoscope.cns.fr/microscope/mage/getInfoLabel.php?id=76484403) | FKP26_RS29180 | [WP_027561771.1](https://www.ncbi.nlm.nih.gov/protein/WP_027561771.1?report=genbank&log$=prottop&blast_rank=1&RID=9R9TSBSE013) | *folD* |
| [VIDU01_170022](https://mage.genoscope.cns.fr/microscope/mage/getInfoLabel.php?id=76485317) | FKP26_RS32990 | [WP_027563577.1](https://www.ncbi.nlm.nih.gov/protein/WP_027563577.1?report=genbank&log$=prottop&blast_rank=1&RID=9RA5MSZD015) | *sga* |
| [VIDU01_200722](https://mage.genoscope.cns.fr/microscope/mage/getInfoLabel.php?id=76486589) | FKP26_RS08875 | [WP_027557222.1](https://www.ncbi.nlm.nih.gov/protein/WP_027557222.1?report=genbank&log$=prottop&blast_rank=1&RID=9RA7KBRK015) | *rpi* |
| [VIDU01_200818](https://mage.genoscope.cns.fr/microscope/mage/getInfoLabel.php?id=76486685) | FKP26_RS47000 | [WP_027557134.1](https://www.ncbi.nlm.nih.gov/protein/WP_027557134.1?report=genbank&log$=prottop&blast_rank=1&RID=9RA93SDU013) | *fdh3A* |
| [VIDU01_270073](https://mage.genoscope.cns.fr/microscope/mage/getInfoLabel.php?id=76487860) | FKP26_RS40110 | [WP_008145224.1](https://www.ncbi.nlm.nih.gov/protein/WP_008145224.1?report=genbank&log$=prottop&blast_rank=1&RID=9RAC5B7K013) | *mdh* |
| [VIDU01_310070](https://mage.genoscope.cns.fr/microscope/mage/getInfoLabel.php?id=76488344) | FKP26_RS10135 | [WP_027559791.1](https://www.ncbi.nlm.nih.gov/protein/WP_027559791.1?report=genbank&log$=prottop&blast_rank=1&RID=9RAE1T2J013) | *pccA* |
| [VIDU01_310356](https://mage.genoscope.cns.fr/microscope/mage/getInfoLabel.php?id=76488630) | FKP26_RS11465 | [WP_027559575.1](https://www.ncbi.nlm.nih.gov/protein/WP_027559575.1?report=genbank&log$=prottop&blast_rank=1&RID=9RAFVVEK013) | *glyA* |
| [VIDU01_320092](https://mage.genoscope.cns.fr/microscope/mage/getInfoLabel.php?id=76489238) | FKP26_RS42420 | [WP_027564329.1](https://www.ncbi.nlm.nih.gov/protein/WP_027564329.1?report=genbank&log$=prottop&blast_rank=1&RID=9RAJN4GW013) | *icl* |
| [VIDU01_420582](https://mage.genoscope.cns.fr/microscope/mage/getInfoLabel.php?id=76490594) | FKP26_RS16500 | [WP_027564911.1](https://www.ncbi.nlm.nih.gov/protein/WP_027564911.1?report=genbank&log$=prottop&blast_rank=1&RID=9RAM4ZUC013) | *purU* |
| [VIDU01_510004](https://mage.genoscope.cns.fr/microscope/mage/getInfoLabel.php?id=76490840) | FKP26_RS46215 | [WP_051378125.1](https://www.ncbi.nlm.nih.gov/protein/WP_051378125.1?report=genbank&log$=prottop&blast_rank=1&RID=9RANN313013) | *ccr* |
| [VIDU01_640301](https://mage.genoscope.cns.fr/microscope/mage/getInfoLabel.php?id=76491678) | FKP26_RS19940 | [WP_027561172.1](https://www.ncbi.nlm.nih.gov/protein/WP_027561172.1?report=genbank&log$=prottop&blast_rank=1&RID=9RAR27ZE013) | *ms* |
| [VIDU01_860194](https://mage.genoscope.cns.fr/microscope/mage/getInfoLabel.php?id=76492497) | FKP26_RS23300 | [WP_027562390.1](https://www.ncbi.nlm.nih.gov/protein/WP_027562390.1?report=genbank&log$=prottop&blast_rank=1&RID=9RAT3925013) | *lutH* |
| VIDU01_860197 | FKP26_RS23315 | [WP_027562387.1](https://www.ncbi.nlm.nih.gov/protein/WP_027562387.1?report=genbank&log$=prottop&blast_rank=1&RID=9RAZ1A1F015) | *frmB* |
| [VIDU01_860199](https://mage.genoscope.cns.fr/microscope/mage/getInfoLabel.php?id=76492502) | FKP26_RS23330 | [WP_027562385.1](https://www.ncbi.nlm.nih.gov/protein/WP_027562385.1?report=genbank&log$=prottop&blast_rank=1&RID=9RB2XEXJ013) | *lutA* |
| [VIDU01_860200](https://mage.genoscope.cns.fr/microscope/mage/getInfoLabel.php?id=76492503) | FKP26_RS23335 | WP_027562384.1 | *lutB* |
| [VIDU01_860201](https://mage.genoscope.cns.fr/microscope/mage/getInfoLabel.php?id=76492504) | FKP26_RS23340 | [WP_027562383.1](https://www.ncbi.nlm.nih.gov/protein/WP_027562383.1?report=genbank&log$=prottop&blast_rank=1&RID=9RB8CBM5015) | *lutE* |
| [VIDU01_860202](https://mage.genoscope.cns.fr/microscope/mage/getInfoLabel.php?id=76492505) | FKP26_RS23345 | [WP_027562382.1](https://www.ncbi.nlm.nih.gov/protein/WP_027562382.1?report=genbank&log$=prottop&blast_rank=1&RID=9RBAAYGV015) | *lutF* |
| [VIDU01_860203](https://mage.genoscope.cns.fr/microscope/mage/getInfoLabel.php?id=76492506) | FKP26_RS23350 | [WP_027562381.1](https://www.ncbi.nlm.nih.gov/protein/WP_027562381.1?report=genbank&log$=prottop&blast_rank=1&RID=9RBCUFMU015) | *lutG* |
| VIDU01_860219 | FKP26_RS23435 | [WP_027562365.1](https://www.ncbi.nlm.nih.gov/protein/WP_027562365.1?report=genbank&log$=prottop&blast_rank=1&RID=9RBJZZT8015) | *exaF* |
| VIDU01_860227 | FKP26_RS23470 | WP_027562358.1 | *xoxF* |
| VIDU01_860228 | FKP26_RS23475 | [WP_027562357.1](https://www.ncbi.nlm.nih.gov/protein/WP_027562357.1?report=genbank&log$=prottop&blast_rank=1&RID=9RBRPYSJ013) | *xoxG* |
| VIDU01_860229 | FKP26_RS23480 | [WP_027562356.1](https://www.ncbi.nlm.nih.gov/protein/WP_027562356.1?report=genbank&log$=prottop&blast_rank=1&RID=9RBTVHE6013) | *frmA* |
| VIDU01_860230 | FKP26_RS23485 | [WP_027562355.1](https://www.ncbi.nlm.nih.gov/protein/WP_027562355.1?report=genbank&log$=prottop&blast_rank=1&RID=9RC1EJH2013) | *gfa* |

Analyses to generate Tables S2 and S3 were conducted in MaGE (47). Because the accession numbers differ between MaGE and NCBI, both sets are provided here for reference.

**Table S2.** Genes involved in Type I and Type II methylotrophy present in *Me.* AM1, *Bs.* 3456, *Bd.* 110, *Sm.* 2011.

| **Gene** | **EC Number** | ***Me.* AM1** | ***Bs.* 3456** | ***Bd.* 110** | ***Sm.* 2011** | **Pathway** |
| --- | --- | --- | --- | --- | --- | --- |
| *ftfL* | [6.3.4.3](https://enzyme.expasy.org/EC/6.3.4.3) | [META1_0329](https://mage.genoscope.cns.fr/microscope/mage/getInfoLabel.php?id=1842996) | - | - | [SM2011_c02728](https://mage.genoscope.cns.fr/microscope/mage/getInfoLabel.php?id=35968420) | H_4_F in *Me.* AM1 |
| *mtdA* | [1.5.1.-](https://enzyme.expasy.org/EC/1.5.1.-) | [META1_1728](https://mage.genoscope.cns.fr/microscope/mage/getInfoLabel.php?id=1844395) | - | - | - | H_4_F in *Me.* AM1 |
| *fch* | [3.5.4.9](https://enzyme.expasy.org/EC/3.5.4.9) | [META1_1729](https://mage.genoscope.cns.fr/microscope/mage/getInfoLabel.php?id=1844396) | - | - | - | H_4_F in *Me.* AM1 |
| *purU* | 3.5.1.10 | - | [VIDU01_420582](https://mage.genoscope.cns.fr/microscope/mage/getInfoLabel.php?id=76490594) | [AAV28_27315](https://mage.genoscope.cns.fr/microscope/mage/getInfoLabel.php?id=78139469) | [SM2011_c03205](https://mage.genoscope.cns.fr/microscope/mage/getInfoLabel.php?id=35969102) | H_4_F Typical |
| *folD* | [1.5.1.5](https://www.brenda-enzymes.org/enzyme.php?ecno=1.5.1.5&UniProtAcc=P24186&OrganismID=2026) | - | [VIDU01_120226](https://mage.genoscope.cns.fr/microscope/mage/getInfoLabel.php?id=76484403) | [AAV28_04035](https://mage.genoscope.cns.fr/microscope/mage/getInfoLabel.php?id=78134937) | [SM2011_c02604](https://mage.genoscope.cns.fr/microscope/mage/getInfoLabel.php?id=35965367) | H_4_F Typical |
| *mtdB* | 1.5.1.- | [META1_1761](https://mage.genoscope.cns.fr/microscope/mage/getInfoLabel.php?id=1844428) | - | - | - | H_4_MPT |
| *mch* | [3.5.4.27](https://enzyme.expasy.org/EC/3.5.4.27) | [META1_1763](https://mage.genoscope.cns.fr/microscope/mage/getInfoLabel.php?id=1844430) | - | - | - | H_4_MPT |
| *glyA* | [2.1.2.1](https://enzyme.expasy.org/EC/2.1.2.1) | [META1_3384](https://mage.genoscope.cns.fr/microscope/mage/getInfoLabel.php?id=1846051) | [VIDU01_310356](https://mage.genoscope.cns.fr/microscope/mage/getInfoLabel.php?id=76488630) | [AAV28_22525](https://mage.genoscope.cns.fr/microscope/mage/getInfoLabel.php?id=78138530) | [SM2011_c01770](https://mage.genoscope.cns.fr/microscope/mage/getInfoLabel.php?id=35966840) | Serine Cycle |
| *sga* | [2.6.1.45](https://enzyme.expasy.org/EC/2.6.1.45) | [META1_1726](https://mage.genoscope.cns.fr/microscope/mage/getInfoLabel.php?id=1844393) | [VIDU01_170022](https://mage.genoscope.cns.fr/microscope/mage/getInfoLabel.php?id=76485317) | [AAV28_27730](https://mage.genoscope.cns.fr/microscope/mage/getInfoLabel.php?id=78139551) | [SM2011_a2139](https://mage.genoscope.cns.fr/microscope/mage/getInfoLabel.php?id=35921827) | Serine Cycle |
| *ppc* | [4.1.1.31](https://enzyme.expasy.org/EC/4.1.1.31) | [META1_1732](https://mage.genoscope.cns.fr/microscope/mage/getInfoLabel.php?id=1844399) | [VIDU01_10600](https://mage.genoscope.cns.fr/microscope/mage/getInfoLabel.php?id=76482967) | [AAV28_11795](https://mage.genoscope.cns.fr/microscope/mage/getInfoLabel.php?id=78136429) | - | Serine Cycle |
| *mdh* | [1.1.1.37](https://enzyme.expasy.org/EC/1.1.1.37) | [META1_1537](https://mage.genoscope.cns.fr/microscope/mage/getInfoLabel.php?id=1844204) | [VIDU01_270073](https://mage.genoscope.cns.fr/microscope/mage/getInfoLabel.php?id=76487860) | [AAV28_29905](https://mage.genoscope.cns.fr/microscope/mage/getInfoLabel.php?id=78139970) | [SM2011_c02479](https://mage.genoscope.cns.fr/microscope/mage/getInfoLabel.php?id=35969262) | Serine Cycle |
| *mtkA* | [6.2.1.9](https://enzyme.expasy.org/EC/6.2.1.9) | [META1_1730](https://mage.genoscope.cns.fr/microscope/mage/getInfoLabel.php?id=1844397) | - | - | - | Serine Cycle |
| *mcl* | [4.1.3.24](https://enzyme.expasy.org/EC/4.1.3.24) | [META1_1733](https://mage.genoscope.cns.fr/microscope/mage/getInfoLabel.php?id=1844400) | - | - | - | Serine Cycle |
| *icl* | 4.1.3.1 | - | [VIDU01_320092](https://mage.genoscope.cns.fr/microscope/mage/getInfoLabel.php?id=76489238) | [AAV28_09190](https://mage.genoscope.cns.fr/microscope/mage/getInfoLabel.php?id=78135913) | [SM2011_c00768](https://mage.genoscope.cns.fr/microscope/mage/getInfoLabel.php?id=35966209) | Glyoxylate Shunt |
| *ms* | 2.3.3.9 | - | [VIDU01_640301](https://mage.genoscope.cns.fr/microscope/mage/getInfoLabel.php?id=76491678) | [AAV28_04320](https://mage.genoscope.cns.fr/microscope/mage/getInfoLabel.php?id=78134994) | [SM2011_c02581](https://mage.genoscope.cns.fr/microscope/mage/getInfoLabel.php?id=35965333) | Glyoxylate Shunt |
| *ccr* | 1.3.1.85 | [META1_0178](https://mage.genoscope.cns.fr/microscope/mage/getInfoLabel.php?id=1842845) | [VIDU01_510004](https://mage.genoscope.cns.fr/microscope/mage/getInfoLabel.php?id=76490840) | - | - | EMC Pathway |
| *pccA* | 6.4.1.3 | [META1_3203](https://mage.genoscope.cns.fr/microscope/mage/getInfoLabel.php?id=1842839) | [VIDU01_310070](https://mage.genoscope.cns.fr/microscope/mage/getInfoLabel.php?id=76488344) | [AAV28_23715](https://mage.genoscope.cns.fr/microscope/mage/getInfoLabel.php?id=78138761) | [SM2011_b20756](https://mage.genoscope.cns.fr/microscope/mage/getInfoLabel.php?id=35938140) | EMC Pathway |
| *mcd* | [1.3.99.10](https://enzyme.expasy.org/EC/1.3.99.10) | [META1_2223](https://mage.genoscope.cns.fr/microscope/mage/getInfoLabel.php?id=1844890) | - | [-](https://mage.genoscope.cns.fr/microscope/mage/getInfoLabel.php?id=78136770) | [-](https://mage.genoscope.cns.fr/microscope/mage/getInfoLabel.php?id=35936258) | EMC Pathway |
| *meaA* | 5.4.99.63 | [META1_0180](https://mage.genoscope.cns.fr/microscope/mage/getInfoLabel.php?id=1842847) | - | [-](https://mage.genoscope.cns.fr/microscope/mage/getInfoLabel.php?id=78136770) | [-](https://mage.genoscope.cns.fr/microscope/mage/getInfoLabel.php?id=35936258) | EMC Pathway |
| *mcmA* | 5.4.99.2 | [META1_5251](https://mage.genoscope.cns.fr/microscope/mage/getInfoLabel.php?id=1845057) | [VIDU01_10717](https://mage.genoscope.cns.fr/microscope/mage/getInfoLabel.php?id=76483084) | [AAV28_12280](https://mage.genoscope.cns.fr/microscope/mage/getInfoLabel.php?id=78136524) | [SM2011_b20757](https://mage.genoscope.cns.fr/microscope/mage/getInfoLabel.php?id=35938142) | EMC Pathway |
| *rpi* | 5.3.1.6 | [META1_2299](https://mage.genoscope.cns.fr/microscope/mage/getInfoLabel.php?id=1844966) | [VIDU01_200722](https://mage.genoscope.cns.fr/microscope/mage/getInfoLabel.php?id=76486589) | [AAV28_15740](https://mage.genoscope.cns.fr/microscope/mage/getInfoLabel.php?id=78137199) | [SM2011_b20371](https://mage.genoscope.cns.fr/microscope/mage/getInfoLabel.php?id=35935313) | RuMP Pathway |
| *hps* | 4.1.2.43 | - | - | - | - | RuMP Pathway |
| *phi* | 5.3.1.27 | - | - | - | - | RuMP Pathway |
| *glpx* | [3.1.3.11](https://enzyme.expasy.org/EC/3.1.3.11) | [META1_0757](https://mage.genoscope.cns.fr/microscope/mage/getInfoLabel.php?id=1843424) | [VIDU01_10119](https://mage.genoscope.cns.fr/microscope/mage/getInfoLabel.php?id=76482486) | [AAV28_18895](https://mage.genoscope.cns.fr/microscope/mage/getInfoLabel.php?id=78137820) | [SM2011_b20202](https://mage.genoscope.cns.fr/microscope/mage/getInfoLabel.php?id=35934916) | RuMP Pathway |
| *tkt* | 2.2.1.1 | [META1_1861](https://mage.genoscope.cns.fr/microscope/mage/getInfoLabel.php?id=1844528) | [VIDU01_10121](https://mage.genoscope.cns.fr/microscope/mage/getInfoLabel.php?id=76482488) | [AAV28_04560](https://mage.genoscope.cns.fr/microscope/mage/getInfoLabel.php?id=78135042) | [SM2011_b20200](https://mage.genoscope.cns.fr/microscope/mage/getInfoLabel.php?id=35934912) | RuMP Pathway |

Genomes from MaGE were queried using EC number (when available) and via BLAST using the sequence of the gene of interest from *Me.* AM1 or *Bs.* 3456 or other organism (indicated in the methods) for the typical H_4_F pathway, glyoxylate shunt, and RuMP pathway. The top hit for each gene is included in the table. In cases where multiple copies are present, only the first, by gene number, is shown for brevity. “-” indicates neither an annotated gene nor a homolog with greater than 50% sequence similarity could be identified.

**Table S3.** Key genes involved in the XoxF-CBB methanol assimilation pathway present in *Me.* AM1, *Bs.* 3456, *Bd.* 110, *Sm.* 2011.

| **Gene** | **EC Number** | ***Me.* AM1** | ***Bs.* 3456** | ***Bd.* 110** | ***Sm.* 2011** | **Description of Gene Product Function** |
| --- | --- | --- | --- | --- | --- | --- |
| *xoxF* | 1.1.2.10 | [META1_1740](https://mage.genoscope.cns.fr/microscope/mage/getInfoLabel.php?id=1844407) | [VIDU01_860227](https://mage.genoscope.cns.fr/microscope/mage/getInfoLabel.php?id=76492530) | [AAV28_28625](https://mage.genoscope.cns.fr/microscope/mage/getInfoLabel.php?id=78139728) | [SM2011_b20173](https://mage.genoscope.cns.fr/microscope/mage/getInfoLabel.php?id=35934862) | Ln-dependent PQQ methanol dehydrogenase. |
| *xoxG* | - | [META1_1741](https://mage.genoscope.cns.fr/microscope/mage/getInfoLabel.php?id=1844408) | [VIDU01_860228](https://mage.genoscope.cns.fr/microscope/mage/getInfoLabel.php?id=76492531) | [AAV28_28630](https://mage.genoscope.cns.fr/microscope/mage/getInfoLabel.php?id=78139729) | [SM2011_b20174](https://mage.genoscope.cns.fr/microscope/mage/getInfoLabel.php?id=35934864) | Cytochrome c. |
| *exaF* | [1.1.99.-](https://enzyme.expasy.org/EC/1.1.99.-) | [META1_1139](https://mage.genoscope.cns.fr/microscope/mage/getInfoLabel.php?id=1843806) | [VIDU01_860219](https://mage.genoscope.cns.fr/microscope/mage/getInfoLabel.php?id=76492522) | [AAV28_28595](https://mage.genoscope.cns.fr/microscope/mage/getInfoLabel.php?id=78139722) | [SM2011_b20173](https://mage.genoscope.cns.fr/microscope/mage/getInfoLabel.php?id=35934862) | Ln-dependent PQQ ethanol dehydrogenase. |
| *gfa* | 4.4.1.22 | [META1_3270](https://mage.genoscope.cns.fr/microscope/mage/getInfoLabel.php?id=1845937) | [VIDU01_860230](https://mage.genoscope.cns.fr/microscope/mage/getInfoLabel.php?id=76492533) | [AAV28_28640](https://mage.genoscope.cns.fr/microscope/mage/getInfoLabel.php?id=78139731) | [SM2011_b20186](https://mage.genoscope.cns.fr/microscope/mage/getInfoLabel.php?id=35934892) | Glutathione-dependent formaldehyde activating enzyme. |
| *frmA* | 1.1.1.284 | - | [VIDU01_860229](https://mage.genoscope.cns.fr/microscope/mage/getInfoLabel.php?id=76492532) | [AAV28_28635](https://mage.genoscope.cns.fr/microscope/mage/getInfoLabel.php?id=78139730) | [SM2011_b20170](https://mage.genoscope.cns.fr/microscope/mage/getInfoLabel.php?id=35934854) | S-(hydroxymethyl)glutathione dehydrogenase. |
| *frmB* | 3.1.2.12 | - | [VIDU01_860197](https://mage.genoscope.cns.fr/microscope/mage/getInfoLabel.php?id=76492500) | [AAV28_28485](https://mage.genoscope.cns.fr/microscope/mage/getInfoLabel.php?id=78139700) | [SM2011_b20171](https://mage.genoscope.cns.fr/microscope/mage/getInfoLabel.php?id=35934856) | S-formylglutathione hydrolase. |
| *fdh1A* | [1.17.1.9](https://www.brenda-enzymes.org/enzyme.php?ecno=1.17.1.9) | [META1_5032](https://mage.genoscope.cns.fr/microscope/mage/getInfoLabel.php?id=1847515) | [VIDU01_110101](https://mage.genoscope.cns.fr/microscope/mage/getInfoLabel.php?id=76483983) | [AAV28_08470](https://mage.genoscope.cns.fr/microscope/mage/getInfoLabel.php?id=78135773) | - | W-Containing formate dehydrogenase. |
| *fdh2A* | [1.17.1.10](https://www.brenda-enzymes.org/enzyme.php?ecno=1.17.1.10) | [META1_4848](https://mage.genoscope.cns.fr/microscope/mage/getInfoLabel.php?id=1847515) | [VIDU01_10810](https://mage.genoscope.cns.fr/microscope/mage/getInfoLabel.php?id=76483177) | [AAV28_12705](https://mage.genoscope.cns.fr/microscope/mage/getInfoLabel.php?id=78136608) | [SM2011_c04444](https://mage.genoscope.cns.fr/microscope/mage/getInfoLabel.php?id=35969205) | Mo-Containing NAD-dependent formate dehydrogenase. |
| *fdh3A* | [1.2.2.1](https://enzyme.expasy.org/EC/1.2.2.1) | [META1_0303](https://mage.genoscope.cns.fr/microscope/mage/getInfoLabel.php?id=1842970) | [VIDU01_200818](https://mage.genoscope.cns.fr/microscope/mage/getInfoLabel.php?id=76486685) | [AAV28_24810](https://mage.genoscope.cns.fr/microscope/mage/getInfoLabel.php?id=78138973) | - | Fe-S formate dehydrogenase. |
| *fdh4A* | - | [META1_2094](https://mage.genoscope.cns.fr/microscope/mage/getInfoLabel.php?id=1844761) | [VIDU01_11139](https://mage.genoscope.cns.fr/microscope/mage/getInfoLabel.php?id=76483506) | [AAV28_40640](https://mage.genoscope.cns.fr/microscope/mage/getInfoLabel.php?id=78142062) | - | Formate dehydrogenase. |
| *prk* | [2.7.1.19](https://enzyme.expasy.org/EC/2.7.1.19) | [META1_0758](https://mage.genoscope.cns.fr/microscope/mage/getInfoLabel.php?id=1843425) | [VIDU01_10120](https://mage.genoscope.cns.fr/microscope/mage/getInfoLabel.php?id=76482487) | [AAV28_09870](https://mage.genoscope.cns.fr/microscope/mage/getInfoLabel.php?id=78136049) | [SM2011_b20201](https://mage.genoscope.cns.fr/microscope/mage/getInfoLabel.php?id=35934914) | Phosphoribulokinase. |
| *cbbL* | 4.1.1.39 | - | [VIDU01_10123](https://mage.genoscope.cns.fr/microscope/mage/getInfoLabel.php?id=76482490) | [AAV28_09885](https://mage.genoscope.cns.fr/microscope/mage/getInfoLabel.php?id=78136052) | [SM2011_b20198](https://mage.genoscope.cns.fr/microscope/mage/getInfoLabel.php?id=35934908) | Large subunit of RuBisCO. |
| *cbbS* | - | - | [VIDU01_10124](https://mage.genoscope.cns.fr/microscope/mage/getInfoLabel.php?id=76482491) | [AAV28_09890](https://mage.genoscope.cns.fr/microscope/mage/getInfoLabel.php?id=78136053) | [SM2011_b20197](https://mage.genoscope.cns.fr/microscope/mage/getInfoLabel.php?id=35934906) | Small subunit of RuBisCO. |
| *cbbX* | - | - | [VIDU01_10125](https://mage.genoscope.cns.fr/microscope/mage/getInfoLabel.php?id=76482492) | [AAV28_09895](https://mage.genoscope.cns.fr/microscope/mage/getInfoLabel.php?id=78136054) | [SM2011_b20196](https://mage.genoscope.cns.fr/microscope/mage/getInfoLabel.php?id=35934904) | RuBisCO regulator. |
| *lutA* | - | [META1_1778](https://mage.genoscope.cns.fr/microscope/mage/getInfoLabel.php?id=1844445) | [VIDU01_860199](https://mage.genoscope.cns.fr/microscope/mage/getInfoLabel.php?id=76492502) | [AAV28_28495](https://mage.genoscope.cns.fr/microscope/mage/getInfoLabel.php?id=78139702) | [SM2011_b20178](https://mage.genoscope.cns.fr/microscope/mage/getInfoLabel.php?id=35934872) | ABC transporter-periplasmic binding component. |
| *lutB* | - | [META1_1779](https://mage.genoscope.cns.fr/microscope/mage/getInfoLabel.php?id=1844446) | [VIDU01_860200](https://mage.genoscope.cns.fr/microscope/mage/getInfoLabel.php?id=76492503) | [AAV28_28500](https://mage.genoscope.cns.fr/microscope/mage/getInfoLabel.php?id=78139703) | [SM2011_b20179](https://mage.genoscope.cns.fr/microscope/mage/getInfoLabel.php?id=35934874) | Exported protein. |
| *lutE* | - | [META1_1782](https://mage.genoscope.cns.fr/microscope/mage/getInfoLabel.php?id=1844449) | [VIDU01_860201](https://mage.genoscope.cns.fr/microscope/mage/getInfoLabel.php?id=76492504) | [AAV28_28505](https://mage.genoscope.cns.fr/microscope/mage/getInfoLabel.php?id=78139704) | [SM2011_b20184](https://mage.genoscope.cns.fr/microscope/mage/getInfoLabel.php?id=35934886) | ABC transporter ATP-binding. |
| *lutF* | - | [META1_1783](https://mage.genoscope.cns.fr/microscope/mage/getInfoLabel.php?id=1844450) | [VIDU01_860202](https://mage.genoscope.cns.fr/microscope/mage/getInfoLabel.php?id=76492505) | [AAV28_28510](https://mage.genoscope.cns.fr/microscope/mage/getInfoLabel.php?id=78139705) | [SM2011_b20185](https://mage.genoscope.cns.fr/microscope/mage/getInfoLabel.php?id=35934888) | ABC transporter membrane component. |
| *lutG* | - | [META1_1784](https://mage.genoscope.cns.fr/microscope/mage/getInfoLabel.php?id=1844451) | [VIDU01_860203](https://mage.genoscope.cns.fr/microscope/mage/getInfoLabel.php?id=76492506) | [AAV28_28515](https://mage.genoscope.cns.fr/microscope/mage/getInfoLabel.php?id=78139706) | [SM2011_b20169](https://mage.genoscope.cns.fr/microscope/mage/getInfoLabel.php?id=35934850) | Exported protein. |
| *lutH* | - | [META1_1785](https://mage.genoscope.cns.fr/microscope/mage/getInfoLabel.php?id=1844452) | [VIDU01_860194](https://mage.genoscope.cns.fr/microscope/mage/getInfoLabel.php?id=76492497) | [AAV28_28470](https://mage.genoscope.cns.fr/microscope/mage/getInfoLabel.php?id=78139697) | - | TonB dependent receptor. |

Genomes from MaGE were queried using EC number (when available) and via BLAST using the sequence of the gene of interest from *Me.* AM1 or *Bs.* 3456 when EC numbers were unavailable. The top hit for each gene is included in the table. In cases where multiple copies are present, only the first, by gene number, is shown for brevity. “-” indicates neither an annotated gene nor a homolog with greater than 50% sequence similarity could be identified.


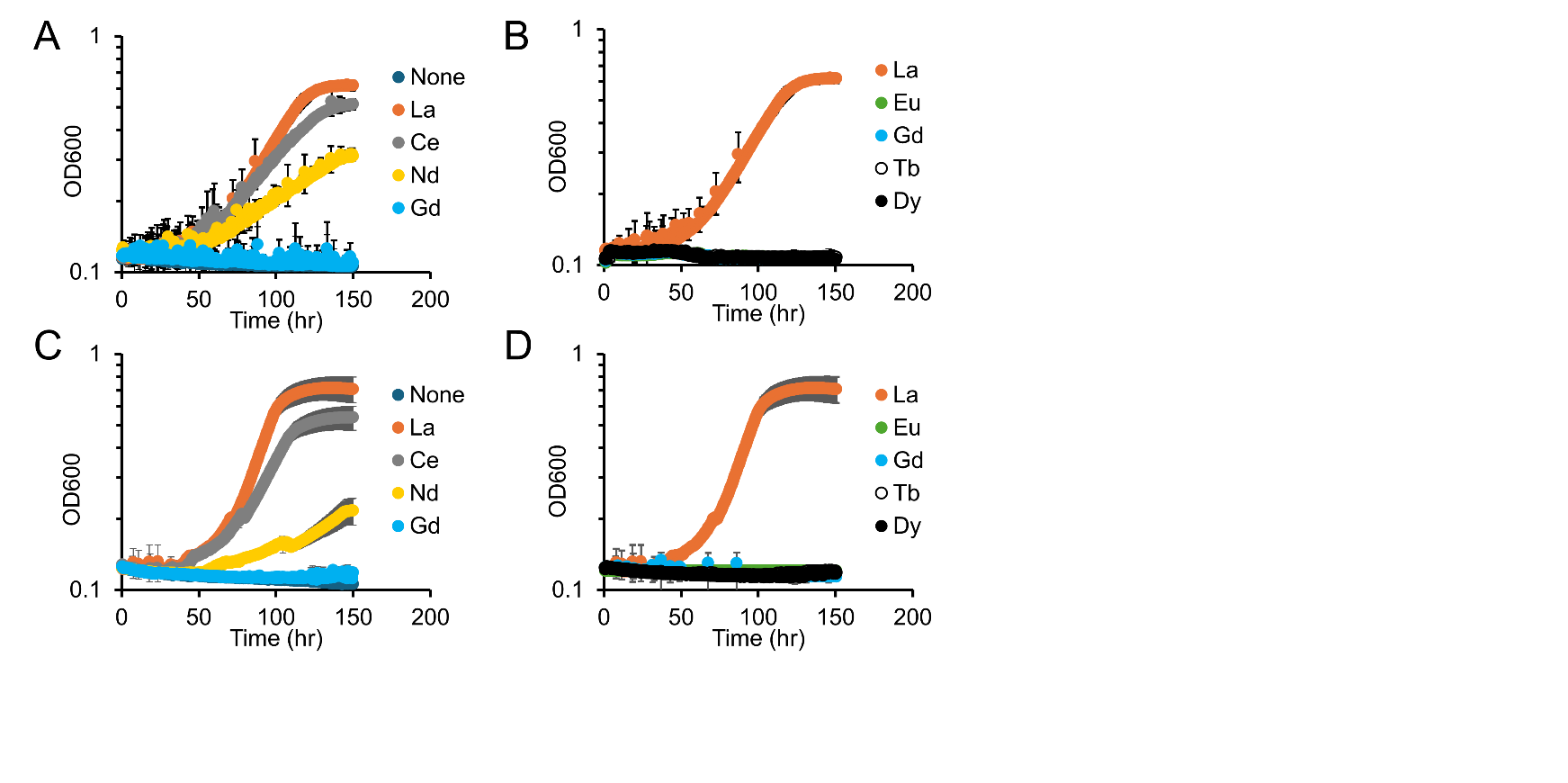


**Fig S1.** Growth of *Bs*. 3456 and *Sm.* 2011 with different Lns. **(A and B)** Growth of *Bs.* 3456 with 50 mM methanol and 10 μM of the indicated lanthanide source. **(C and D)** Growth of *Sm.* 2011 with 50 mM methanol and 10 μM of the indicated lanthanide source. For all panels, error bars indicate the standard deviation of 3 replicates.


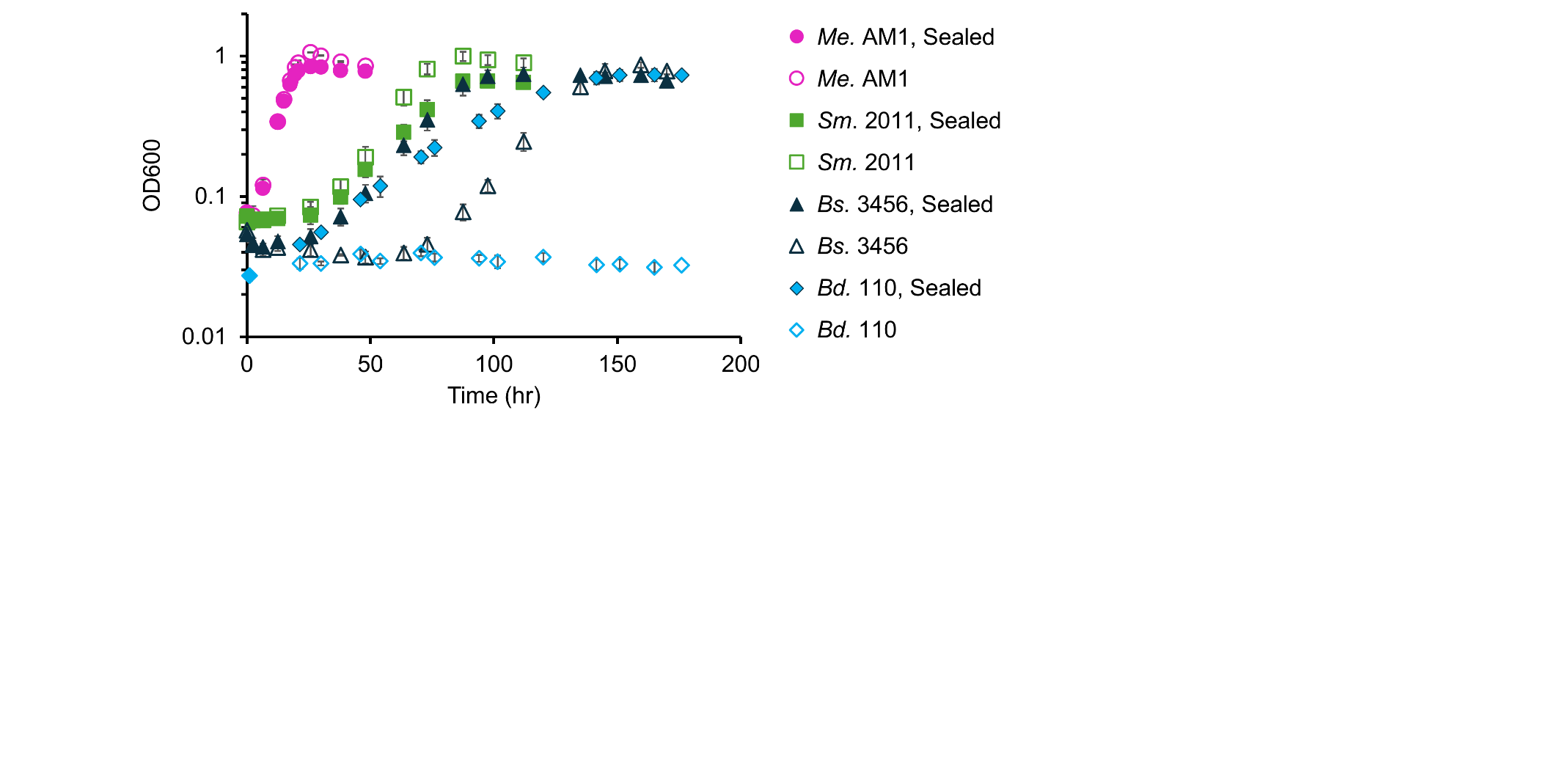


**Fig S2:** Growth in sealed (filled symbol) vs foil-covered (open symbol) Balch-style tubes facilitates growth of *Bd.* 110 (diamonds) and increases growth rate of *Bs.* 3456 (triangles) but does not increase the growth rate of *Me.* AM1 (circles) or *Sm.* 2011 (squares) with methanol. All samples were grown with 50 mM methanol with 10 μM LaCl_3_. Error bars indicate the standard deviation of 3 replicates.


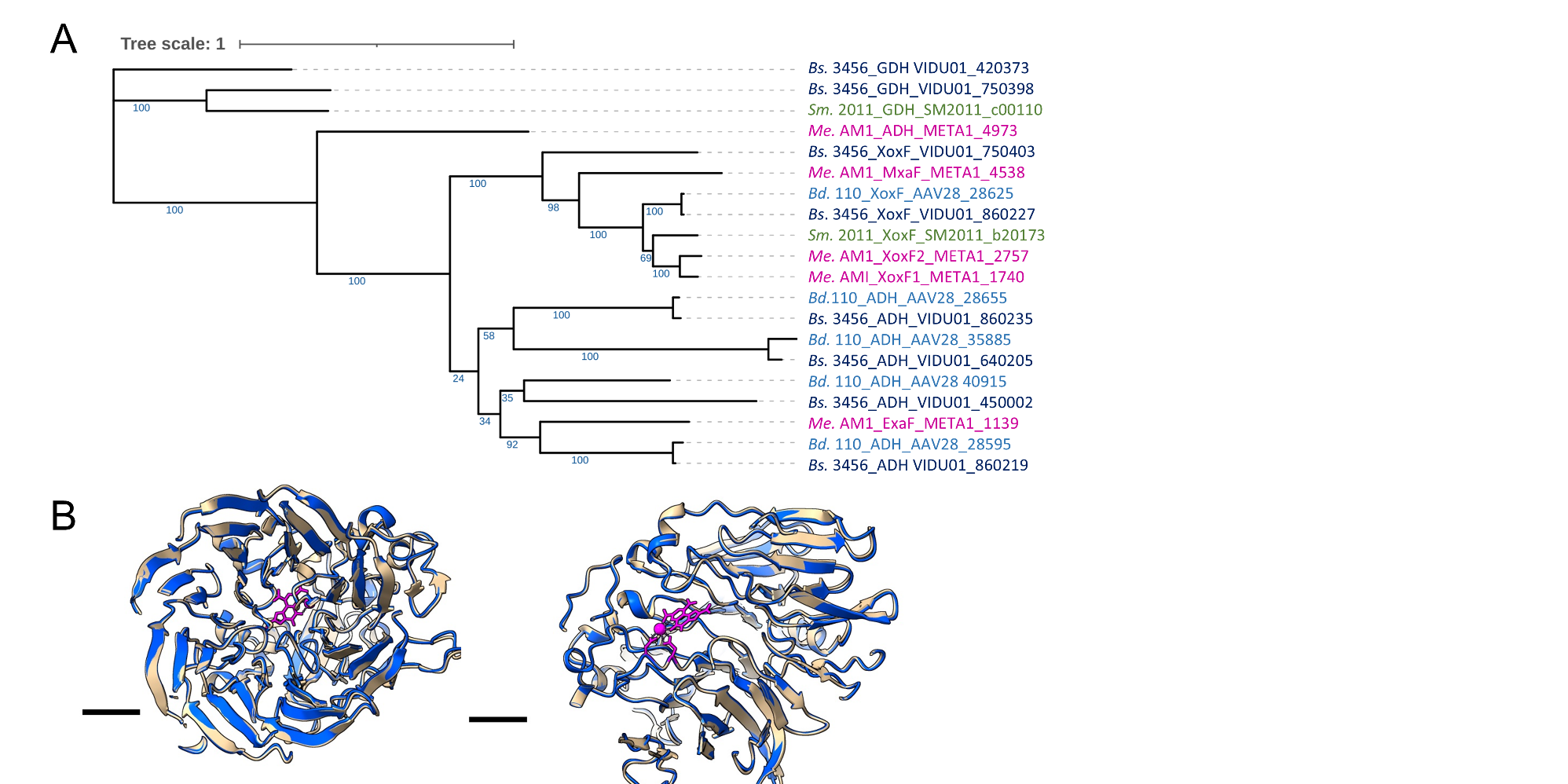


**Fig S3.** Sequence and structure relationships of alcohol dehydrogenases found in the strains of interest**. (A)** BLAST search using XoxF1 from AM1 as the query reveals multiple potential Ln-dependent proteins encoded in each genome of interest. Strain names, gene product annotations, and accession numbers from MaGE are provided and color-coded according to the strain. *Bradyrhizobium diazoefficiens.* USDA 110 light blue, *Bradyrhizobium* sp. USDA 3456 dark blue, *Sinorhizobium meliloti 2011* green, *Methylobacterium extorquens* AM1 pink. All but AAV28_24975, META1_4973, META1_4538, and VIDU01_450002 are predicted to be Ln-dependent based on the presence of an ‘additional Ln-binding Asp’ in the active site. Bootstrap values are the result of 1,000 ultrafast calculations in IQTree (49). **(B)** Comparison of overall structure and Ln-binding residues between the *Bs.* 3456 XoxF (VIDU01_860227) modeled with >90% confidence in Alpha Fold3 (blue) and the crystal structure of XoxF1 from *Me.* AM1 (tan) with PQQ and Ce present (PDB 6OC6). PQQ, Ce, and the catalytic and Ln-binding Asps from both models are highlighted in pink. Scale bar = 10Å.


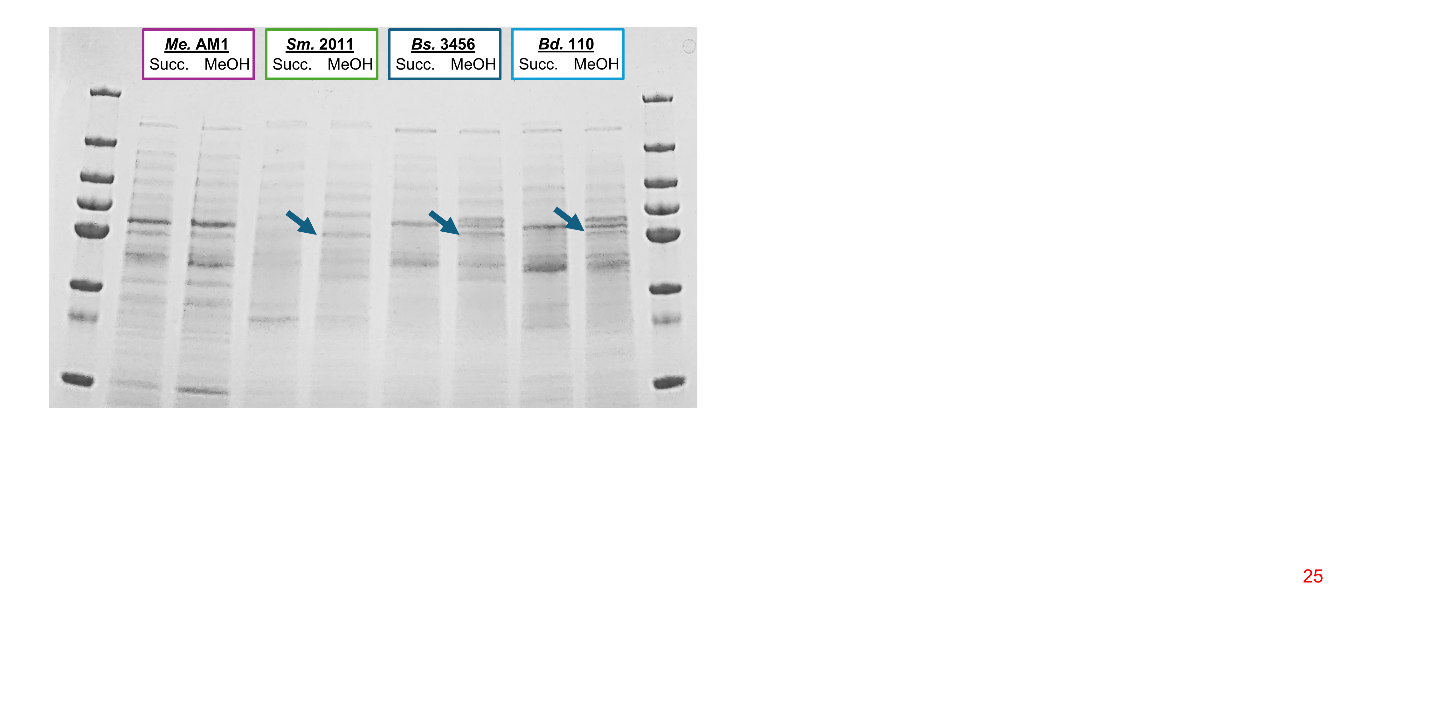


**Fig S4.** SDS-PAGE of cell lysates used for 1-D proteomics. Clarified supernatants from the indicated strains grown with either succinate + La (Succ.) or methanol + La (MeOH) were analyzed via 4-12% gradient SDS-PAGE with a PageRuler Plus Prestained protein ladder. Blue arrows indicate the approximately 55 kDa bands sent for analysis. Each sample well contains approximately 20 μg total protein.
